# Tumor-derived small extracellular vesicles facilitate omental metastasis of ovarian cancer by triggering activation of mesenchymal stem cells

**DOI:** 10.1186/s12964-023-01413-9

**Published:** 2024-01-17

**Authors:** Lanqing Gong, Guoqing Li, Xiaoqing Yi, Qing Han, Qiulei Wu, Feiquan Ying, Lu Shen, Ying Cao, Xiaoli Liu, Lingling Gao, Wenhan Li, Zehua Wang, Jing Cai

**Affiliations:** 1 Department of Obstetrics and Gynecology, Union Hospital, Tongji Medical College, Huazhong University of Science and Technology, Wuhan, 430022 China; 2 Department of Obstetrics and Gynecology, The First College of Clinical Medical Science, China Three Gorges University, Yichang, Hubei 443000 China; 3 Department of Gynecology and Obstetrics, The Affiliated Suzhou Hospital of Nanjing Medical University, Suzhou Municipal Hospital, Gusu School, Nanjing Medical University, Suzhou, Jiangsu China; 4 Department of Obstetrics and Gynecology, The Central Hospital of Wuhan, Tongji Medical College, Huazhong University of Science and Technology, Wuhan, 430030 China

**Keywords:** Ovarian cancer, Adipose-derived mesenchymal stem cell, Small extracellular vesicle, miR-320a, Integrin α7

## Abstract

**Background:**

Omental metastasis is the major cause of ovarian cancer recurrence and shortens patient survival, which can be largely attributed to the dynamic evolution of the fertile metastatic microenvironment driven by cancer cells. Previously, we found that adipose-derived mesenchymal stem cells (ADSCs) undergoing a phenotype shift toward cancer-associated fibroblasts (CAFs) participated in the orchestrated omental premetastatic niche for ovarian cancer. Here, we aim to elucidate the underlying mechanisms.

**Methods:**

Small extracellular vesicles were isolated from ovarian cancer cell lines (ES-2 and its highly metastatic subline, ES-2-HM) and patient ascites using ultracentrifugation. Functional experiments, including Transwell and EdU assays, and molecular detection, including Western blot, immunofluorescence, and RT–qPCR, were performed to investigate the activation of ADSCs in vitro. High-throughput transcriptional sequencing and functional assays were employed to identify the crucial functional molecules inducing CAF-like activation of ADSCs and the downstream effector of miR-320a. The impact of extracellular vesicles and miR-320a-activated ADSCs on tumor growth and metastasis was assessed in subcutaneous and orthotopic ovarian cancer xenograft mouse models. The expression of miR-320a in human samples was evaluated using in situ hybridization staining.

**Results:**

Primary human ADSCs cocultured with small extracellular vesicles, especially those derived from ES-2-HM, exhibited boosted migration, invasion, and proliferation capacities and elevated α-SMA and FAP levels. Tumor-derived small extracellular vesicles increased α-SMA-positive stromal cells, fostered omental metastasis, and shortened the survival of mice harboring orthotopic ovarian cancer xenografts. miR-320a was abundant in highly metastatic cell-derived extracellular vesicles, evoked dramatic CAF-like transition of ADSCs, targeted the 3′-untranslated region of integrin subunit alpha 7 and attenuated its expression. miR-320a overexpression in ovarian cancer was associated with omental metastasis and shorter survival. miR-320a-activated ADSCs facilitated tumor cell growth and omental metastasis. Depletion of integrin alpha 7 triggered CAF-like activation of ADSCs in vitro.

Video Abstract

**Conclusions:**

miR-320a in small extracellular vesicles secreted by tumor cells targets integrin subunit alpha 7 in ADSCs and drives CAF-like activation, which in turn facilitates omental metastasis of ovarian cancer.

**Graphical Abstract:**

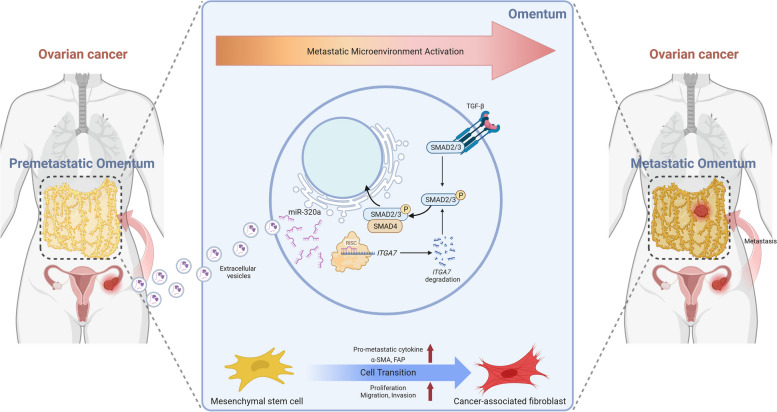

**Supplementary Information:**

The online version contains supplementary material available at 10.1186/s12964-023-01413-9.

## Background

Ovarian cancer (OC) is the second most common cause of gynecological cancer deaths in females worldwide [1, 2]. The majority of OC patients have developed distant metastases when diagnosed, resulting in a dismal 5-year survival rate of 20–41% [3]. Therefore, elucidating the detailed mechanisms of OC metastasis is a critical issue for reducing OC-related deaths. During the development and progression of metastasis, the dynamic evolution of the metastatic microenvironment is essential for the settlement and colonization of tumor cells in target organs [4]. Several strategies targeting such metastatic microenvironments to prevent tumor metastasis have been reported [5], but ambiguity about the mechanisms underlying the dynamic changes in metastatic microenvironments limits their clinical use. Therefore, it is necessary to explore the cells and molecular drivers triggering dynamic changes in the metastatic microenvironment.

Mesenchymal stem cells (MSCs) are a subpopulation of pluripotent stem cells with the ability to self-renew and undergo multipotent differentiation [6] and actively participate in tumor-associated inflammation, immunosuppression, tumor growth, angiogenesis, and tumor metastasis, playing a crucial role in the tumor microenvironment [7]. The omentum, a major metastatic target site for OC, is enriched in adipose tissue and resides in numerous adipose-derived mesenchymal stem cells (ADSCs) [8, 9]. Accumulating studies have shown that active ADSCs promote and maintain the malignant phenotype of tumors by altering the proteomic profile of OC cells and driving metabolic reprogramming through paracrine mechanisms [10–12]. A recent report found that one type of adipocyte-derived fibroblasts characterized by both MSCs and cancer-associated fibroblasts (CAFs) was found in the omentum to activate OC cells, causing them to display enhanced proliferative properties and migratory capacity [13]. In addition, we previously demonstrated that omental ADSCs could be activated by OC cells to acquire a CAF-like phenotype, which helps OC to form a carcinogenic metastatic microenvironment in the omentum to promote the colonization and growth of tumor cells [9]. However, it is unclear how cancer cells trigger such CAF-like activation of MSCs, which hampers the strategy for targeting MSCs to overcome OC metastasis.

The reciprocal interaction between tumor cells and stromal cells of targeted organs is largely dependent on cell-secreted soluble factors, including cytokines, chemokines and extracellular vesicles [14, 15]. Compared with other tumor cell-secreted factors, extracellular vesicles have membrane structures that make them more resistant to degradation and abundant with biological components, including nucleic acids, proteins and lipids, enabling them to be an important mediator for the transport of biological information in the metastatic microenvironment [16]. Recently, cancer-secreted small extracellular vesicles (sEV) have been reported to be responsible for tumor microenvironment remodeling, such as inflammation [17], immunosuppression [18], vascular permeability [19], and extracellular matrix remodeling [20], to maintain tumor growth, stemness, drug tolerance and metastasis potential. Furthermore, we have previously elucidated that OC cells secrete oncogenic sEV and deliver them to the omentum, establishing a premetastatic microenvironment to support the arrival and colonization of tumor cells [21]. Cho JA et al. also reported that exosomes derived from OC contributed to generating tumor-associated myofibroblasts from MSCs in tumor stroma [22]. These studies imply that OC-derived extracellular vesicles play a crucial cancer-promoting role in activating ADSCs and shaping the metastatic microenvironment. Therefore, further exploration is warranted into the molecular mechanism through which tumor cells induce ADSC activation in the metastatic site through delivering extracellular vesicles to facilitate tumor metastasis.

In this work, we identified that OC-derived sEV transfer miR-320a to omental ADSCs and thereby triggers CAF-like activation and consequently facilitates omental metastasis. In addition, we demonstrate that miR-320a directly targets integrin subunit alpha 7 (*ITGA7*) and mobilizes the TGF-beta pathway to elicit this activation, which highlights the functional and molecular significance of the miR-320a/ITGA7/TGF-beta axis in the metastatic microenvironment. This study unravels the underlying mechanism by which OC cells induce CAF-like activation of ADSCs, providing a theoretical rationale for the development of MSC-based therapeutic strategies for OC metastasis.

## Results

### Tumor-derived small extracellular vesicles drive CAF-like activation of ADSCs

**Fig. 1:**
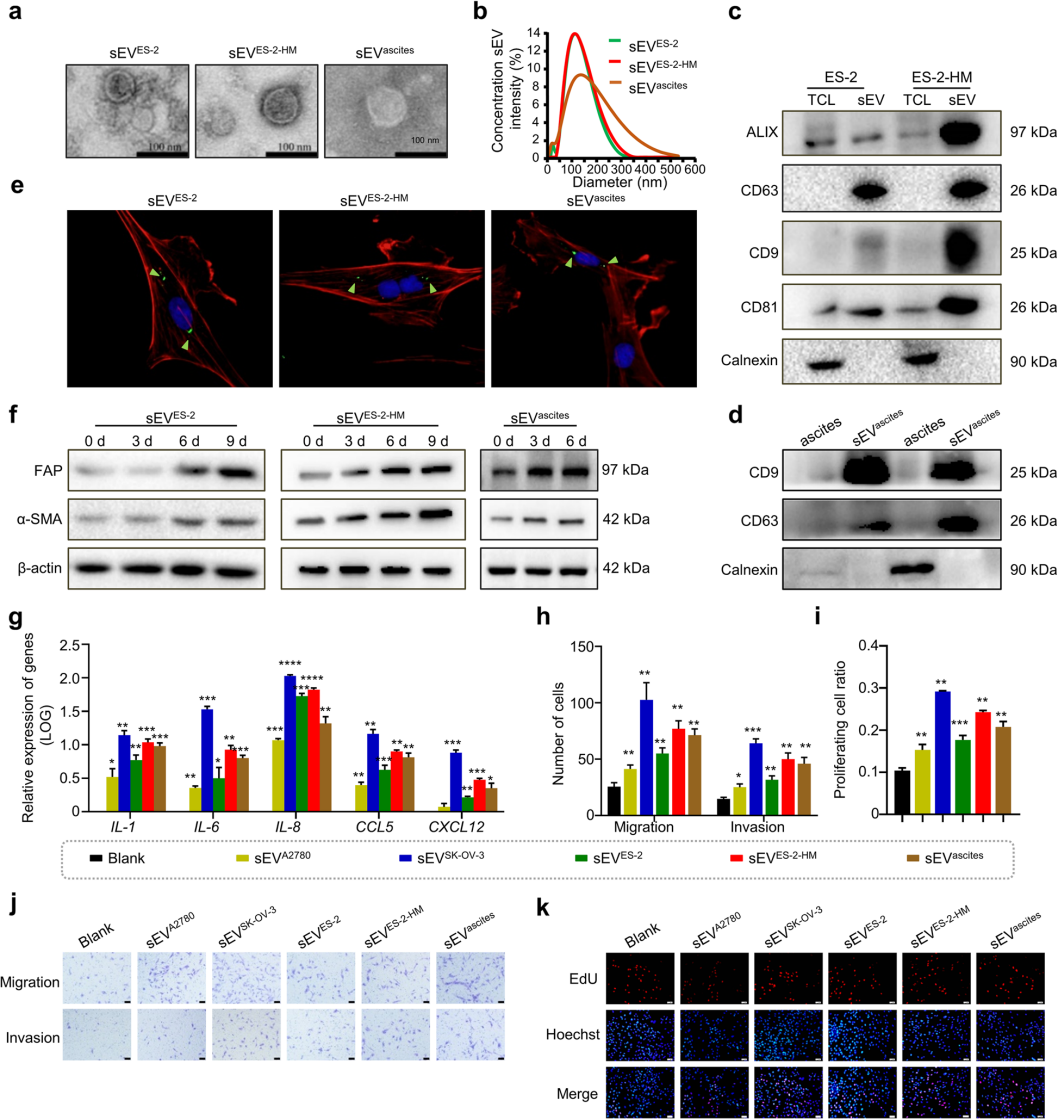
Small extracellular vesicles derived from ovarian cancer cells induce the activation of ADSCs. **a** Microscopic morphological structure of small extracellular vesicles (sEV) derived from ES-2 (sEV^ES-2^), ES-2-HM cells (sEV^ES-2-HM^) and ascites of ovarian cancer patients (sEV^ascites^) photographed by transmission electron microscopy. Scale bar, 100 nm. **b** Size characterization of sEV^ES-2^, sEV^ES-2-HM^ and sEV^ascites^ measured using nanoparticle tracking analysis. **c** Levels of positive markers ALIX, CD9, CD63 and CD81 and negative marker Calnexin of sEV in total cell lysates (TCL) and sEV derived from ES-2 and ES-2-HM cells examined by Western blot. **d** Levels of CD9, CD63 and Calnexin in ascites and sEV^ascites^ examined by Western blot. **e** Representative images of adipose-derived mesenchymal stem cell (ADSC) uptake of PKH67-labeled sEV. Red staining represents the cytoskeleton, green staining represents the sEV, and blue staining represents the nucleus. **f** The levels of cancer-associated fibroblast (CAF) markers (FAP and α-SMA) in ADSCs cocultured with sEV^ES-2^, sEV^ES-2-HM^ or sEV^ascites^ for different durations (0, 3, 6, or 9 days) were examined by Western blot. **g** Expression of CAF-expressing cytokines (*IL-1*, *IL-6*, *IL-8*, *CCL5* and *CXCL12*) in ADSCs cocultured with sEV derived from A2780 (sEV^A2780^), SK-OV-3 (sEV^SK-OV-3^), sEV^ES-2^, sEV^ES-2-HM^ or sEV^ascites^ detected by RT–qPCR. Statistical results show alterations in the migration, invasion (**h**), and proliferation (**i**) capacity of ADSCs cocultured with sEV^A2780^, sEV^SK-OV-3^, sEV^ES-2^, sEV^ES-2-HM^ or sEV^ascites^. **j** Representative images of migration and invasion assays demonstrate the migratory and invasive abilities of ADSCs under the indicated coculture conditions. Scale bar, 50 μm. **k** Representative images of the EdU assay demonstrate the proliferative capacity of ADSCs under the indicated coculture conditions. Scale bar, 100 μm. * *P* < 0.05, ** *P* < 0.01, *** *P* < 0.001, **** *P* < 0.0001

To investigate the effect of tumor-derived sEV on ADSC activation, sEV derived from three OC cell lines, A2780, SK-OV-3 and ES-2, and ES-2-HM, a highly metastatic ES-2 subline, and ascites from OC patients were isolated and characterized (Fig. 1a-d, Supplementary Fig. 1a). Immunofluorescence demonstrated that these extracellular vesicles could be taken up by ADSCs (Fig. 1e, Supplementary Fig. 1b). Western blot and immunofluorescence analyses demonstrated that extracellular vesicles derived from OC cell lines and the ascites of OC patients, increased the levels of α-SMA and FAP in ADSCs, commonly recognized markers for CAFs (Fig. 1f, Supplementary Fig. 1c-f). Moreover, the expression of various CAF-associated cytokines, including *IL-1*, *IL-6*, *IL-8*, *CCL5* and *CXCL12*, in ADSCs was significantly elevated after coincubation with sEV derived from tumor cells and ascites, as detected by RT–qPCR (Fig. 1g). Furthermore, Transwell and EdU assays showed that these sEV also strengthened the proliferative, migratory and invasive capacity of ADSCs compared to the blank control (Fig. 1h-k, Supplementary Fig. 1 g, h). Intriguingly, we found that the more invasive SK-OV-3 and ES-2-HM cells (Supplementary Fig. 2) induced this CAF-like activation to a greater extent than A2780 and ES-2 cells. In addition, tumor cell-derived conditioned medium (CM) containing abundant extracellular vesicles also increased α-SMA, FAP, *IL-1*, *IL-6*, *IL-8*, *CCL5* and *CXCL12* expression and enhanced the proliferation, motility and invasion of ADSCs (Supplementary Fig. 3). These results indicate that tumor-derived sEV promote CAF-like activation of ADSCs.

### Small extracellular vesicles facilitate tumor metastasis by triggering CAF-like activation of ADSCs

**Fig. 2:**
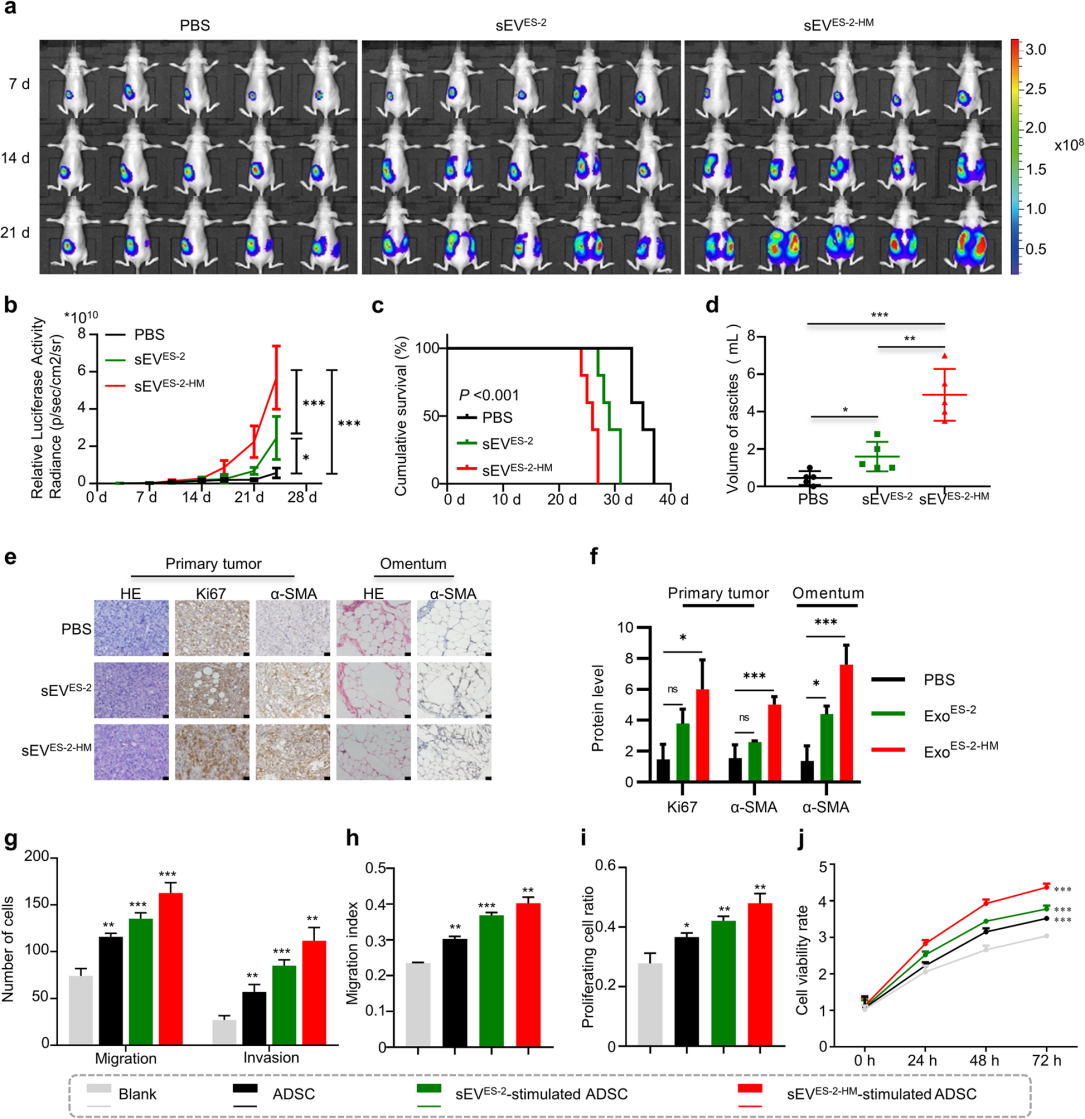
Small extracellular vesicles derived from ovarian cancer cells facilitate tumor metastasis by triggering ADSC activation. **a** Dynamic monitoring of bioluminescence signals in luciferase-labeled ES-2 ovarian orthotopic engraftment tumor-bearing mice injected intraperitoneally with PBS, small extracellular vesicles (sEV) derived from ES-2 (sEV^ES-2^) or sEV derived from ES-2-HM (sEV^ES-2-HM^) by in vivo animal imaging. **b** The statistics of total fluorescence intensity of the above mice at different times. **c** Survival curve analysis of the above mice. **d** Statistical results of the volume of ascites when the above mice were sacrificed. **e** Representative immunohistochemical images of Ki67 and α-SMA levels in tumors and omentum of ovarian orthotopic engraftment tumor-bearing mice after PBS, sEV^ES-2^ or sEV^ES-2-HM^ injection. Scale bar, 20 μm. **f** Statistical results of tumoral and omental Ki67 and α-SMA levels in the above mice. Statistical results of migration, invasion (**g**-**h**), proliferation (**i**) and viability (**j**) of ES-2 cells cocultured with adipose-derived mesenchymal stem cells (ADSCs), sEV^ES-2^-stimulated ADSCs or sEV^ES-2-HM^-stimulated ADSCs. ns, no significance, * *P* < 0.05, ** *P* < 0.01, *** *P* < 0.001

Nude mice with ovarian orthotopic xenografts were injected intraperitoneally with PBS, sEV derived from ES-2 (sEV^ES-2^) or ES-2-HM cells (sEV^ES-2-HM^) (Fig. 2a). Bioluminescence in vivo imaging revealed that sEV^ES-2-HM^ significantly accelerated tumor growth compared to PBS and sEV^ES-2^ 14 days after sEV injection (Fig. 2b). Moreover, the survival of mice in the sEV^ES-2-HM^ group was shorter than that in the PBS and sEV^ES-2^ groups (Fig. 2c). Mice injected intraperitoneally with sEV^ES-2-HM^ also developed significantly increased volumes of ascites at execution (Fig. 2d). In addition, these extracellular vesicles, especially sEV^ES-2-HM^, increased the number of Ki67-positive tumor cells, representing proliferating cells (Fig. 2e, f). In the stroma, sEV^ES-2-HM^ also significantly increased the number of intratumoral and omental α-SMA-positive cells compared with PBS and sEV^ES-2^ (Fig. 2e, f). Then, tumor cells were cocultured with CM from ADSCs, sEV^ES-2^-stimulated ADSCs or sEV^ES-2-HM^-stimulated ADSCs. Transwell and Wound healing assays demonstrated that tumor cells cocultured with ADSCs, particularly sEV-stimulated ADSCs, possessed increased mobility and invasiveness (Fig. 2g, h, Supplementary Fig. 4a, b). Furthermore, sEV^ES-2-HM^-stimulated ADSCs dramatically enhanced the proliferation and viability of tumor cells compared to the blank control, as detected by EdU, colony formation and cell viability assays (Fig. 2i, j, Supplementary Fig. 4c, d). Collectively, the above results suggest that sEV from cancer cells, especially those with high metastatic potential, contribute to the CAF-like activation of ADSCs for tumor growth and omental metastasis.

### miR-320a in small extracellular vesicles promotes CAF-like activation of ADSCs

**Fig. 3:**
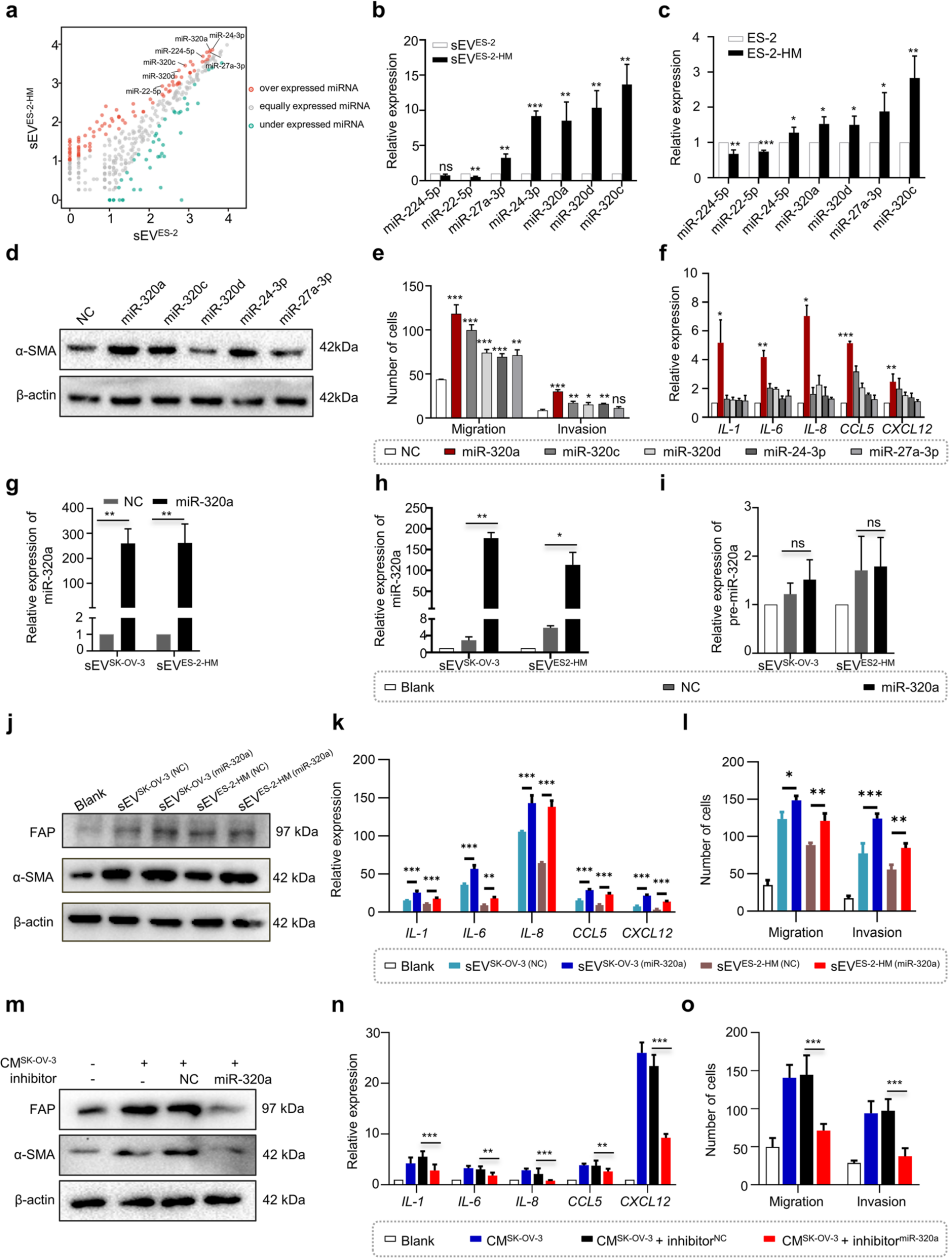
Highly metastatic ovarian cancer cells secrete miR-320a-enriched small extracellular vesicles triggering ADSC activation. **a** Differential miRNA profiles between small extracellular vesicles (sEV) derived from ES-2 (sEV^ES-2^) and ES-2-HM (sEV^ES-2-HM^) were detected by next-generation sequencing. Red dots represent overexpressed miRNAs, gray dots represent nondifferentially expressed miRNAs, and green dots represent underexpressed miRNAs. **b** Differential expression of seven miRNAs (miR-224-5p, miR-22-5p, miR-27a-3p, miR-24-3p, miR-320a, miR-320d, and miR-320c) between sEV^ES-2^ and sEV^ES-2-HM^ examined by RT–qPCR. **c** Differential expression of seven miRNAs between ES-2 and ES-2-HM cells examined by RT–qPCR. **d** The α-SMA levels of ADSCs overexpressing miR-320a, miR-320c, miR-320d, miR-24-3p, or miR-27a-3p detected by Western blot. **e** Statistical results of migration and invasion assays display the motility and invasiveness of ADSCs that individually overexpressed the five miRNAs. **f** Expression of CAF-associated cytokines (*IL-1*, *IL-6*, *IL-8*, *CCL5* and *CXCL12*) in ADSCs that individually overexpressed the five miRNAs examined by RT–qPCR. **g** The expression of miR-320a in the sEV derived from ES-2-HM and SK-OV-3 cells that overexpress miR-320a and its negative control counterpart detected by RT–qPCR. Expression of (**h**) mature miR-320a and (**i**) premiR-320a in ADSCs cocultured with miR-320a-overexpressing sEV and negative controls detected by RT–qPCR. **j** α-SMA and FAP levels of ADSCs cocultured with ovarian cancer cell-derived sEV overexpressing miR-320a (sEV^SK-OV-3 (miR-320a)^ and sEV^ES-2-HM (miR-320a)^) and the negative control (sEV^SK-OV-3 (NC)^ and sEV^ES-2-HM (NC)^). **k** Expression of CAF-associated cytokines in ADSCs cocultured with sEV^SK-OV-3 (miR-320a)^, sEV^ES-2-HM (miR-320a)^, sEV^SK-OV-3 (NC)^, or sEV^ES-2-HM (NC)^. **l** Statistical results of migration and invasion assays display the migration and invasion ability of ADSCs cocultured with sEV^SK-OV-3 (miR-320a)^, sEV^ES-2-HM (miR-320a)^, sEV^SK-OV-3 (NC)^, or sEV^ES-2-HM (NC)^. **m** α-SMA and FAP levels in ADSCs with inhibition of miR-320a expression in the presence of SK-OV-3 conditioned medium (CM^SK-OV-3^). **n** Expression of CAF-associated cytokines in ADSCs with inhibition of miR-320a expression in the presence of CM^SK-OV-3^. **o** Statistical results of migration and invasion assays display the migration and invasion ability of ADSCs with inhibition of miR-320a expression in the presence of CM^SK-OV-3^. ns, no significance, * *P* < 0.05, ** *P* < 0.01, *** *P* < 0.001

To screen critical functional molecules that induce the differentiation of ADSCs in OC cell-derived sEV, high-throughput sequencing was performed to compare the miRNA transcriptional profiles between sEV^ES-2^ and sEV^ES-2-HM^. Fifty-two upregulated and seventy-seven downregulated miRNAs in sEV^ES-2-HM^ were identified, of which the seven most dramatically upregulated miRNAs, i.e., miR-224-5p, miR-22-5p, miR-27a-3p, miR-24-3p, miR-320a, miR-320d, and miR-320c, were detected by RT–qPCR (Fig. 3a, b). In addition, miR-27a-3p, miR-24-3p, miR-320a, miR-320d, and miR-320c were significantly overexpressed in ES-2-HM cells compared with ES-2 cells (Fig. 3c); thus, these five miRNAs were chosen for further experiments. Overexpression of individual miR-24-3p, miR-320a, and miR-320c by mimic transfection in ADSCs resulted in elevated expression of α-SMA detected by Western blot (Fig. 3d, Supplementary Fig. 5a). Moreover, these miRNAs significantly enhanced the migration and invasion abilities of ADSCs, as evidenced by Transwell assays (Fig. 3e, Supplementary Fig. 5b). They also increased the expression of CAF-related cytokines, as detected by RT-qPCR (Fig. 3f).

Among these five miRNAs, miR-320a exhibited a prominent contribution to the activation of ADSCs and was chosen for further experiments. RT–qPCR showed an increase in miR-320a expression in ADSCs, induced by CM and sEV derived from OC cells (Supplementary Fig. 5c-e) and sEV derived from ascites of OC patients (Supplementary Fig. 5f). The sEV from OC cells overexpressing miR-320a were overloaded with miR-320a compared to the negative control (Fig. 3g). In addition, while miR-320a-enriched sEV increased mature miR-320a expression in ADSCs (Fig. 3h), it did not affect pre-miR-320a expression (Fig. 3i). This suggests that sEV elevate miR-320a expression in ADSCs through delivery, not by inducing transcription. Moreover, Western blot and RT-qPCR analyses showed that the sEV overloaded with miR-320a significantly elevated FAP, α-SMA, *IL-1*, *IL-6*, *IL-8*, *CCL5* and *CXCL12* expression (Fig. 3j, k). Transwell assays demonstrated that coincubation of ADSCs with miR-320a-rich sEV also enhanced the migration and invasiveness of ADSCs (Fig. 3l, Supplementary Fig. 5 g). The expression of FAP, α-SMA, and CAF-associated cytokines, as well as the migratory and invasive properties of ADSCs with miR-320a inhibition, were reduced after coculture with CM from SK-OV-3 cells compared to coculture with CM from SK-OV-3 cells only (Fig. 3m-o, Supplementary Fig. 5 h). In summary, these findings underscore the role of tumor-derived sEV in triggering CAF-like activation of ADSCs via transport of miR-320a.

### miR-320a is associated with tumor metastasis and poor prognosis in ovarian cancer patients

**Fig. 4:**
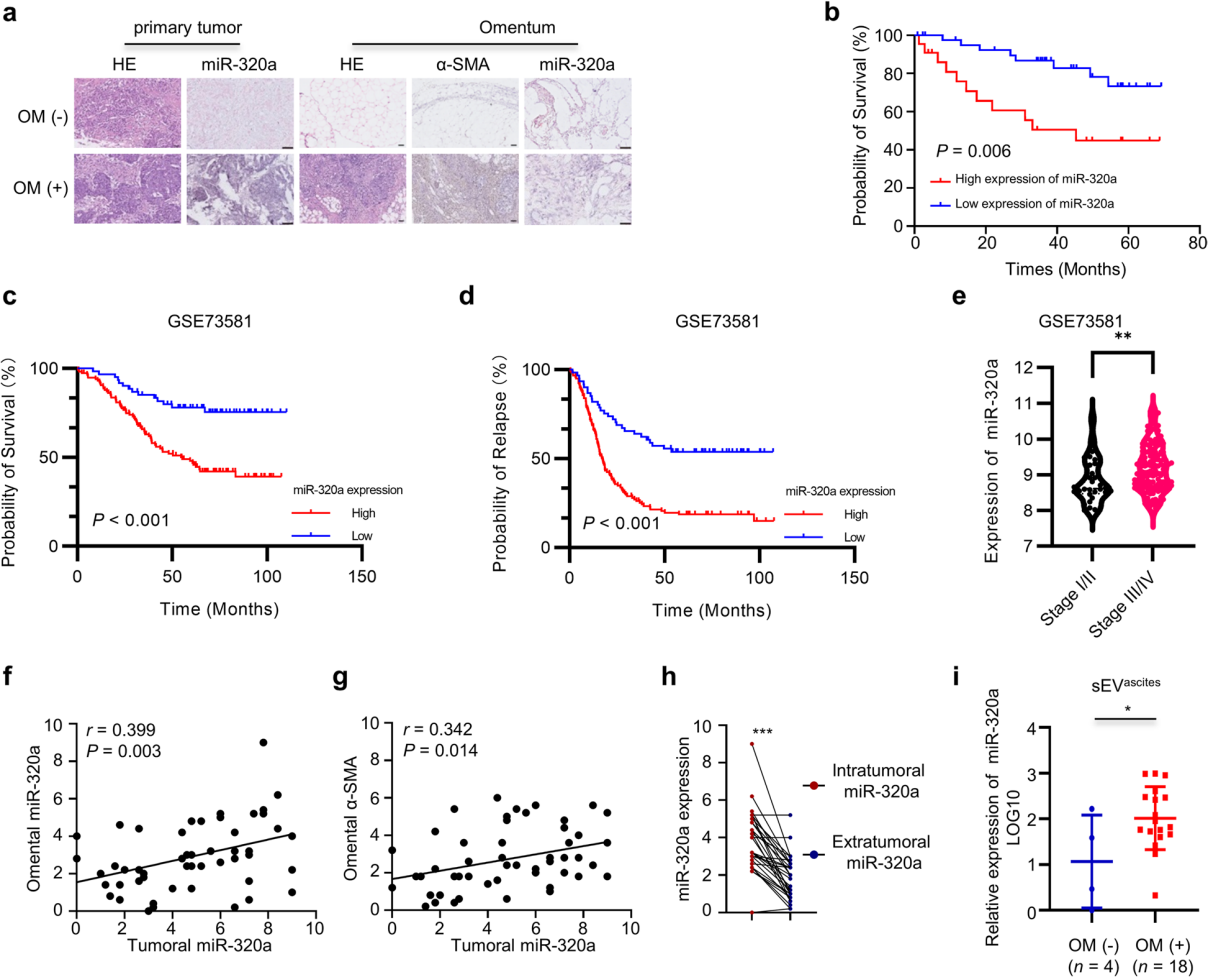
miR-320a is associated with worse prognosis and activation of the omental microenvironment. **a** Representative images of in situ hybridization for miR-320a and immunohistochemistry for α-SMA staining of ovarian cancer. Scale bar, 50 μm. **b** Survival curve of ovarian cancer patients with high and low expression of miR-320a in our hospital. **c** Survival curve of overall survival for ovarian cancer patients with high and low miR-320a expression in the GSE73581 dataset. **d** Curve of time to relapse for ovarian cancer patients with high and low miR-320a expression in the GSE73581 dataset. **e** The expression of miR-320a in stage I/II and stage III/IV ovarian cancer patients in the GSE73581 dataset. **f** Correlation between tumoral miR-320a expression and omental miR-320a expression. **g** Correlation between tumoral miR-320a expression and omental α-SMA levels. **h** Expression of miR-320a between intratumoral and extratumoral mesenchymal components in the metastatic omentum of ovarian cancer patients. **i** Expression of miR-320a in ascites-derived sEV (sEV^ascites^) of ovarian cancer patients with and without omental metastases detected by RT–qPCR. * *P* < 0.05, ** *P* < 0.01, *** *P* < 0.001.

The clinical significance of miR-320a was investigated using OC samples. In situ hybridization staining revealed the presence of miR-320a in both tumor cells and the surrounding stroma (Fig. 4a). The high expression of miR-320a in OC was associated with advanced FIGO stage (*P* = 0.018), omentum metastasis (*P* = 0.021) and lymph node metastasis (*P* = 0.033). Similarly, the high expression of miR-320a in the omental stroma was associated with high-grade serous ovarian cancer (*P* = 0.023), advanced FIGO stage (*P* = 0.004), and omentum metastasis (*P* < 0.001) (Table 1). miR-320a overexpression in OC was associated with shorter survival of patients (Fig. 4b). Furthermore, in silico analysis of the GSE73581 dataset showed a correlation between high miR-320a expression and shorter overall survival times, as well as time to relapse (Fig. 4c, d). Also, miR-320a expression was higher in advanced tumors compared to early tumors (Fig. 4e). We further found that miR-320a expression was positively correlated between the tumor and omentum (Fig. 4f), and there was also a significant positive correlation between tumoral miR-320a expression and omental α-SMA levels (Fig. 4 g). In addition, within the omentum with metastases, intratumoral mesenchyme exhibited higher miR-320a expression than extratumoral mesenchyme (Fig. 4 h). The ascites-derived sEV from patients with omental metastases were also enriched in miR-320a (Fig. 4i). Altogether, both clinical sample staining and bioinformatic analyses suggest a close association between high miR-320a expression and worse prognosis, short survival, and activation of the omental microenvironment in OC patients.

**Table 1:** The association between miR-320a expression and clinicopathological features in ovarian cancer patients

Variables	miR-320a expression in ovarian cancer				miR-320a expression in omentum			
	*n*	Low expression	High expression	*P* value	*n*	Low expression	High expression	*P* value
**Age**				0.691				0.661
≤ 49	24	14	10		23	17	6	
> 49	39	26	13		31	20	11	
**Pathology**				0.314				0.023
HGSOC	46	27	19		38	22	16	
Non-HGSOC	17	13	4		16	15	1	
**FIGO stage**				0.018				0.004
I – II	18	16	2		16	16	0	
III – IV	45	24	21		38	21	17	
**Omentum metastasis**				0.021				< 0.001
Absent	27	22	5		23	22	1	
Present	36	18	18		31	15	16	
**Lymph node metastasis**				0.033				0.084^*^
Absent	49	35	14		41	31	10	
Present	14	5	9		13	6	7	
**Appendix metastasis**				0.081^*^				0.056^*^
Absent	52	36	16		44	33	11	
Present	11	4	7		10	4	6	

* fisher test

*FIGO* International Federation of Gynecology and Obstetrics, *HGSOC* high-grade serous ovarian cancer

### miR-320a in small extracellular vesicles facilitates tumor metastasis by promoting the activation of ADSCs

**Fig. 5:**
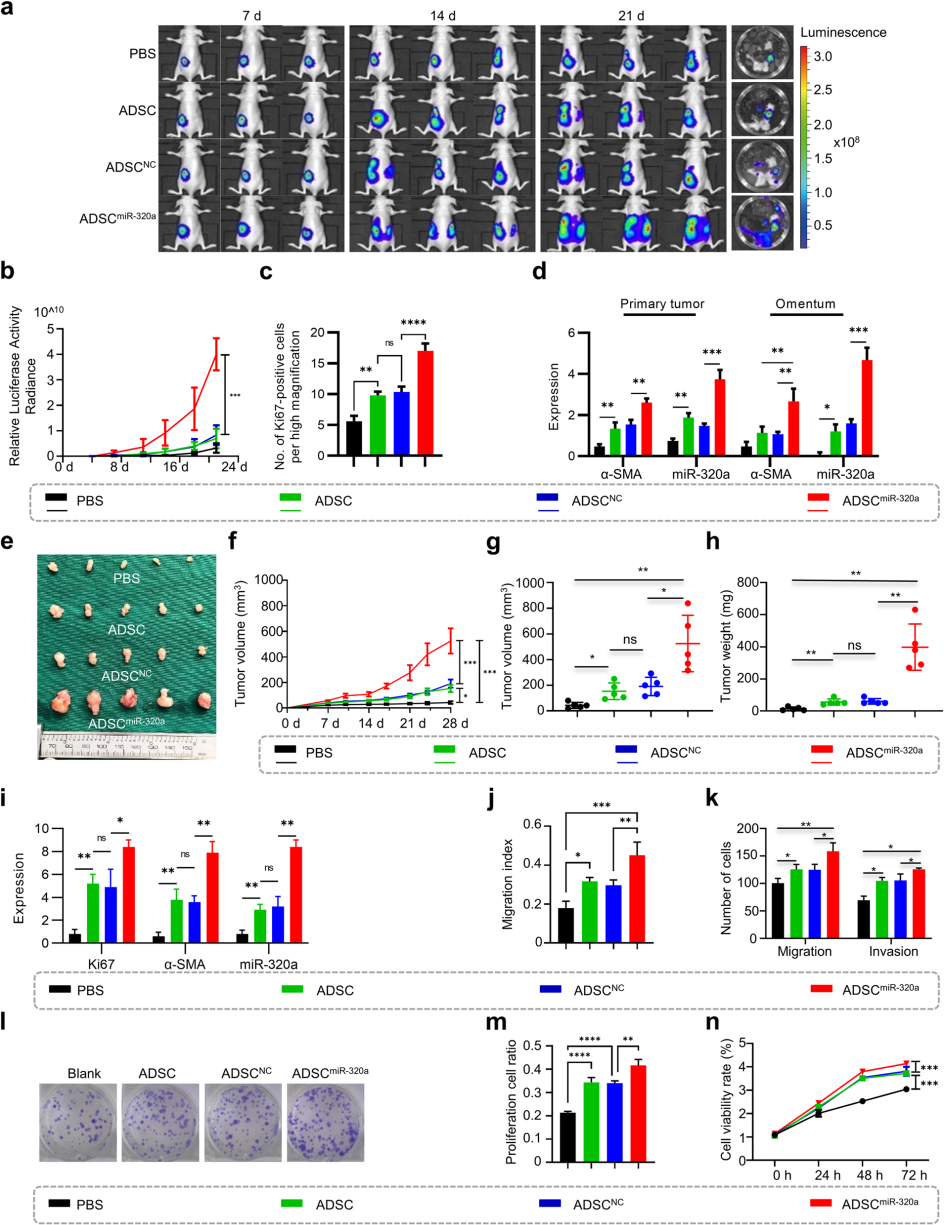
miR-320a facilitates ovarian cancer progression via the activation of ADSCs. **a** Dynamic monitoring of bioluminescence signals in luciferase-labeled ES-2 ovarian orthotopic engraftment tumor-bearing mice injected intraperitoneally with PBS, adipose-derived mesenchymal stem cells (ADSCs), negative control ADSCs (ADSCs^NC^) or ADSCs overexpressing miR-320a (ADSCs^miR-320a^) by in vivo animal imaging. **b** Statistics of the total fluorescence intensity of the above mice at different times. **c** Statistical results of Ki67-positive cell count in tumor foci of ovarian orthotopic engraftment tumor-bearing mice. **d** Statistical results of miR-320a and α-SMA expression in tumor foci and omentum of ovarian orthotopic engraftment tumor-bearing mice. **e** Gross appearance of nude mice with subcutaneous xenograft tumors of SK-OV-3 mixed with PBS, ADSCs, ADSCs^NC^, or ADSCs^miR-320a^. **f** The volume change of subcutaneous tumor growth described above. **g** Tumor volume of the above mice when executed. **h** Tumor weight of the above mice when executed. **i** The expression of α-SMA, Ki67, and miR-320a in SK-OV-3 subcutaneous tumors. **j** Statistical results of motility of ES-2 cells indirectly cocultured with PBS, ADSCs, ADSCs^NC^, or ADSCs^miR-320a^ detected by Wound healing assay. **k** Statistical results of migration and invasion assays display the motility and invasiveness of ES-2 cells indirectly cocultured with PBS, ADSCs, ADSCs^NC^, or ADSCs^miR-320a^. **l** The results of the colony formation assay of SK-OV-3 cells coincubated with conditioned media from ADSCs, ADSCs^NC^, or ADSCs^miR-320a^. **m** Statistical results of the proliferation of SK-OV-3 cells coincubated with conditioned media of ADSCs, ADSCs^NC^, or ADSCs^miR-320a^ detected by EdU assays. **n** The viability of SK-OV-3 cells coincubated with conditioned media of ADSCs, ADSCs^NC^, or ADSCs^miR-320a^ detected by CCK8 assay. ns, no significance, * *P* < 0.05, ** *P* < 0.01, *** *P* < 0.001, **** *P* < 0.0001

To determine whether ADSC activation induced by sEV delivery of miR-320a contributes to OC progression, mice with orthotopic ovarian transplantation were injected intraperitoneally with PBS, ADSCs, negative control ADSCs (ADSCs^NC^) or ADSCs overexpressing miR-320a (ADSCs^miR-320a^) (Fig. 5a, Supplementary Fig. 6a). Bioluminescence in vivo imaging demonstrated significantly accelerated tumor growth in the ADSCs^miR-320a^ group compared to the ADSCs^NC^ and PBS groups (Fig. 5b). Moreover, there was a significant increase in the number of intratumoral Ki67-positive cells in the ADSCs^miR-320a^ group (Fig. 5c, Supplementary Fig. 6b). Immunohistochemical staining demonstrated that ADSCs^miR-320a^ significantly elevated intratumoral and omental α-SMA levels compared to PBS and ADSCs^NC^ (Fig. 5d, Supplementary Fig. 6b, c). In addition, SK-OV-3 cells mixed with PBS, ADSCs, ADSCs^NC^ or ADSCs^miR-320a^ were injected subcutaneously into nude mice (Fig. 5e). Notably, ADSCs, particularly those overexpressing miR-320a, significantly accelerated tumor growth as evidenced by increased tumor size, weight, and number of Ki67-positive cells (Fig. 5f-i), along with elevated intratumoral α-SMA levels (Fig. 5i, Supplementary Fig. 6d). Moreover, in vitro assays revealed that OC cells indirectly cocultured with ADSCs^miR-320a^ acquired strengthened migratory and invasive properties (Fig. 5j, k, Supplementary Fig. 7a, b), along with accelerated proliferation and improved cell viability (Fig. 5 l-n, Supplementary Fig. 7c). These findings demonstrate that miR-320a-induced CAF-like activation of ADSCs drives tumor growth and metastasis.

### miR-320a targets *ITGA7* to activate the TGF-β pathway for triggering ADSC activation

**Fig. 6:**
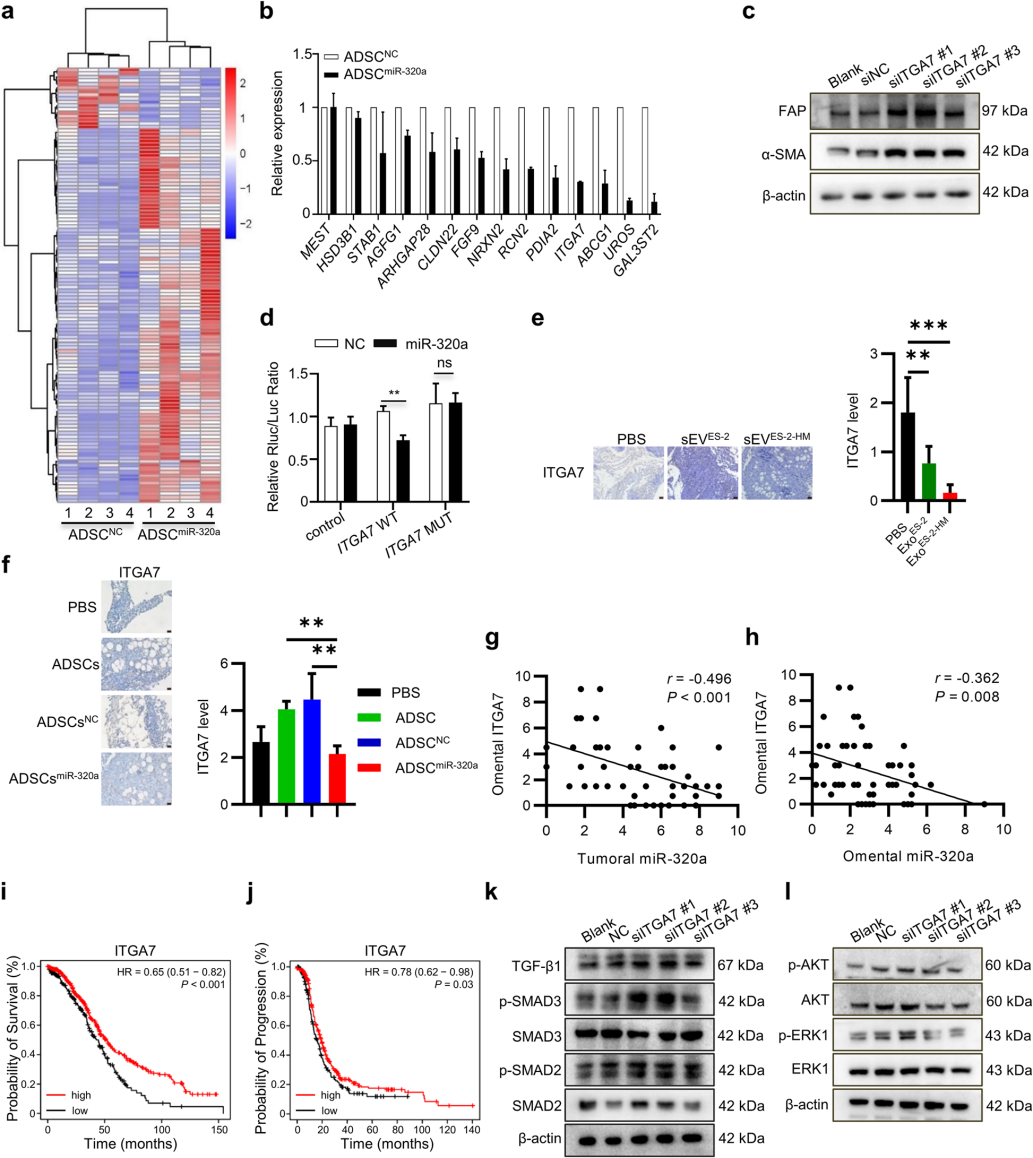
miR-320a triggers the TGF-β/SMAD2/SMAD3 pathway by targeting *ITGA7*. **a** Differential mRNA transcriptional profiles between negative control ADSCs (ADSCs^NC^) and ADSCs overexpressing miR-320a (ADSCs^miR-320a^) examined by high-throughput sequencing. **b** The expression of 14 differentially expressed genes identified by RNA sequencing between ADSCs^NC^ and ADSCs^miR-320a^ detected by RT–qPCR. **c** The α-SMA and FAP levels of ADSCs with *ITGA7* silencing detected by Western blot. **d** Luciferase activity in ADSCs transfected with the Renilla luciferase plasmid with *ITGA7* mutation detected by dual luciferase assay. **e** Representative immunohistochemical images (left) and statistical results (right) of ITGA7 levels in omentum of ovarian orthotopic engraftment tumor-bearing mice after PBS, sEV derived from ES-2 cells (sEV^ES-2^), or sEV derived from ES-2-HM cells (sEV^ES-2-HM^) injection. Scale bar, 20 μm. **f** Representative immunohistochemical images (left) and statistical results (right) of ITGA7 staining in the omentum of ovarian orthotopic engraftment tumor-bearing mice injected intraperitoneally with PBS, adipose-derived mesenchymal stem cells (ADSCs), negative control ADSCs (ADSCs^NC^), or ADSCs overexpressing miR-320a (ADSCs^miR-320a^). Scale bar, 20 μm. **g** Correlation between tumoral miR-320a expression and omental ITGA7 levels. **h** Correlation between omental miR-320a expression and omental ITGA7 levels. **i** Curve of overall survival for ovarian cancer patients with high and low *ITGA7* expression assessed on Kaplan–Meier Plotter. **j** Curve of progression-free survival for ovarian cancer patients with high and low *ITGA7* expression assessed on Kaplan–Meier Plotter. **k** TGF-β1, SMAD2, SMAD3, phosphorylated SMAD2 (p-SMAD2), and phosphorylated SMAD3 (p-SMAD3) levels in *ITGA7*-silenced ADSCs detected by Western blot. **l** AKT, ERK1, phosphorylated AKT (p-AKT), and phosphorylated ERK1 (p-ERK1) levels in *ITGA7*-silenced ADSCs detected by Western blotting. ns, no significance, ** *P* < 0.01, *** *P* < 0.001

To delve deeper into the molecular mechanism of activation of ADSCs driven by miR-320a, differential transcriptional profiles between ADSCs^NC^ and ADSCs^miR-320a^ were assessed by high-throughput sequencing. We identified 105 upregulated and 14 downregulated genes in ADSCs^miR-320a^ compared to ADSCs^NC^ (Fig. 6a). Since miRNAs destabilize target mRNA transcripts as one of essential mechanism for post-transcriptional regulation [23], we selected the 14 down-regulated genes for subsequent experiments and validated them using RT-qPCR. The results showed that six genes, *RCN2*, *PDIA2*, *ITGA7*, *ABCG1*, *UROS*, and *GAL3ST2*, were significantly downregulated in ADSCs^miR-320a^ compared to ADSCs^NC^ (Fig. 6b). Then, the expression of these six individual genes was silenced in ADSCs (Supplementary Fig. 8). Silencing *ITGA7* and *UROS* led to a significant increase in α-SMA and FAP levels in ADSCs (Fig. 6c, Supplementary Fig. 9a-c). In contrast, the silencing of *RCN2*, *PDIA2*, *ABCG1*, and *GAL3ST2* activated ADSCs but to a negligible effect (Supplementary Fig. 9d-k). The dual luciferase reporter assays showed that miR-320a significantly diminished the luciferase activity of the *ITGA7* wild-type vector (Fig. 6d), but no significant alteration was observed in the *UROS* wild-type vector (Supplementary Fig. 10a), indicating that miR-320a directly targets the 3′ untranslated regions of *ITGA7* but not that of *UROS*. We also found that omental ITGA7 levels were reduced in ovarian orthotopically transplanted mice injected intraperitoneally with sEV^ES-2-HM^ (Fig. 6e). In addition, intraperitoneal injection of ADSCs^miR-320a^ also significantly diminished ITGA7 levels in the omentum of mice orthotopically transplanted with ovarian tumors (Fig. 6f). Furthermore, OC and omentum staining for ITGA7 and miR-320a demonstrated a negative correlation between tumoral miR-320a expression and omental ITGA7 levels (Fig. 6 g), as well as between omental miR-320a expression and omental ITGA7 levels (Fig. 6 h). Kaplan–Meier survival analyses showed that low expression of *ITGA7* in OC was associated with short overall survival and progression-free survival of patients (Fig. 6i, j). These findings highlight the clinical significance of ITGA7 and reveal that miR-320a directly targets *ITGA7* to induce CAF-like activation of ADSCs. It has been reported that mobilization of the PI3K-AKT, ERK-MAPK, and TGF-β signaling pathways is responsible for the activation and differentiation of MSCs [24, 25]. Western blot analysis was used to detect the effects of ITGA7 and UROS on these signaling pathways. The results revealed that *ITGA7* silencing increased the phosphorylation of SMAD3 and activated the TGF-β pathway (Fig. 6 k) but failed to activate the ERK-MAPK and PI3K-AKT pathways. (Fig. 6 l). However, the silencing of *UROS* only activated the ERK-MAPK pathways without affecting the TGF-β pathway (Supplementary Fig. 10b, c). Taken together, these results demonstrate that miR-320a targets *ITGA7* to activate the TGF-β pathway, thereby triggering CAF-like activation of ADSCs.

## Discussion

As a dynamic system orchestrated by intercellular communication, the stepwise development of the tumor microenvironment is tightly bound to tumor progression and metastasis [4]. It has been shown that activation of MSCs creates a fertile metastatic microenvironment, thereby fostering tumor migration and growth [26, 27], especially in OC [8, 28]. Here, we highlight tumor-derived sEV as a critical mediator for CAF-like activation of omental ADSCs and elucidate the central role of the miR-320a/ITGA7/TGF-beta axis in facilitating such activation and OC metastasis. These results uncover a novel molecular mechanism underlying the acquisition of a procarcinogenic phenotype for ADSCs triggered by tumor cells.

The tumor microenvironment evolves spatially and temporally during tumor development, resulting in heterogeneity of the tumor microenvironment, which challenges therapeutic strategies targeting the tumor microenvironment. MSCs are pluripotent stem cells originating from the mesoderm with self-renewal capacity and multidirectional differentiation potential and are engaged in directing the evolution of the entire tumor microenvironment [29], contributing to the heterogeneity of the tumor microenvironment. Distinct active phases of MSCs exert contrasting actions on tumors. On the one hand, native MSCs exert antitumor activity by controlling angiogenesis, regulating the cell cycle and inducing apoptosis [29]. On the other hand, tumor-associated MSCs are specifically reprogrammed by tumor signaling to exhibit a tumor-promoting phenotype and support tumor progression and aggressiveness [30, 31]. Several stimuli have been shown to affect stem cell differentiation, including both physical forces (e.g., matrix stiffness, electrical stimulation) and biochemical molecules (e.g., growth factors, extracellular vesicles) [32, 33]. Li HJ et al. found that cancer cells induce prostaglandin E secretion by MSCs through interleukin-1, which evokes activation of β-catenin signaling and cancer stem cell formation [34]. Zheng Z et al. indicated that Th2 cytokines induce and maintain C3 expression to evoke the acquisition of metastasis-promoting properties in lung MSCs [35]. Here, we found that tumor-derived sEV are critical biological contributors to inducing CAF-like activation of ADSCs. These diverse and complex stimuli elicit heterogeneity of MSCs, which further leads to heterogeneity of the tumor microenvironment.

Extracellular vesicles are considered potent vehicles between tumor cells and stromal cells because of their essential role in intercellular communication and engagement in shaping the tumor microenvironment [36]. Compared with other biological signals (e.g., growth factors and chemokines), extracellular vesicles are structurally stable, and their contents are more resistant to degradation by extracellular bioenzymes, making them more suitable for educating the distant microenvironment. Although MSC-derived extracellular vesicles regulate many cancer behaviors, such as proliferation [37], metastasis [38] and epithelial-mesenchymal transition [39], and have been extensively studied, few studies have explored the effects of extracellular vesicles from tumor cells modifying MSCs. This study bridges this research gap and illuminates the extracellular vesicle-dependent crosstalk circuit between tumor cells and MSCs.

Numerous studies have suggested that aberrant miR-320a expression is observed in various cancers and functions in tumor progression [40–43]. In OC, miR-320a promotes the growth and invasion of tumor cells by targeting Ras association domain family member 8 [44], and the overexpression of miR-320a manifests a worse prognosis and high risk of metastasis [45]. Likewise, our data showed that miR-320a overexpression is associated with shorter survival, malignant pathological type and distant metastasis in OC patients. Furthermore, we demonstrated that miR-320a is overexpressed in highly metastatic tumor cells and is incorporated into sEV for activation of ADSCs in metastatic target organs, contributing to evolution of the metastatic microenvironment. In contrast, Huang Y et al. found that miR-320a derived from extracellular vesicles inhibits tumor cell proliferation and suppressed migration, invasion and angiogenesis, demonstrating a tumor-suppressive effect [46]. This likely results from the spatiotemporal heterogeneity of extracellular vesicles exerting cellular communication functions. In other words, extracellular vesicles enriched with miR-320a exert diametrically opposite effects on different receptor cell types in the tumor microenvironment at different stages of tumor progression. Interestingly, other members of the miR-320 family, including miR-320c and miR-320d, were found to be elevated in the sEV derived from highly metastatic cancer cells. These members also exhibited the capability to activate ADSCs, though less effectively than miR-320a. Prior research has indicated potential associations between miR-320b and miR-320d and platinum resistance in OC patients [47]. Furthermore, plasma exosomal miR-320d has shown promise as a noninvasive and effective diagnostic biomarker for OC [48]. These observations underscore the significant role of the miR-320 family in the progression of OC. Given that these members of the miR-320 family are miRNAs with highly similar sequences originating from different precursors, it is plausible that this sequence could play a pivotal role in the progression of OC.

Integrins are prime cell adhesion receptors that empower tumor cells to proliferate unrestrictedly, invade across tissue boundaries, and survive in foreign microenvironments by serving as signaling molecules and mechanotransducers and mediating cell migration [49]. Numerous studies have demonstrated that integrins in cancer cells are important for tumor metastasis [50–52], and therapeutic approaches based on blocking integrin signaling inhibit the metastatic dissemination of OC [53]. However, there are no clinical trial agents targeting integrin α7, which is mainly attributed to the lack of investigations on integrin α7 in cancer. Although ITGA7 has been identified as highly expressed in OC compared to the normal ovarian surface epithelium and its overexpression indicates a poor prognosis [54], we revealed that ITGA7 functions as a tumor suppressor and restricts the malignant transition of ADSCs. This suggests that integrin α7 might function distinctly in diverse cells within the ovarian cancer microenvironment. Therefore, systematic targeting for integrin α7 regimens might be a strategy that attends to one thing and loses another. In addition, we found that *ITGA7* silencing activated the TGF-β pathway through increased phosphorylation of SMAD3. Ying F et al. also indicated that integrin α7 inhibits the maturation of TGF-β1 [55]. These results suggest that integrin α7 is a critical molecule limiting TGF-β-mediated tumor progression. The functional effects and mechanisms by which integrin α7 restricts TGF-β pathway activity warrant further study.

## Conclusions

Highly metastatic tumor cells suppress ITGA7 via small extracellular vesicle delivery of miR-320a to trigger the CAF-like activation of ADSCs, thereby boosting tumor metastasis. Elucidating the rationale for such sEV-mediated communication between tumor cells and ADSCs inducing malignant transition of the microenvironment provides new insights for developing potential MSC-based therapeutic targets for OC metastasis.

## Methods

### Cell culture

The OC cell Lines A2780, ES-2, SK-OV-3, and OVCAR-4 were purchased from the China Center for Type Culture Collection (Wuhan University). The highly metastatic ES-2 subline ES-2-HM was constructed in our laboratory [56]. Briefly, ES-2 cells were implanted in the ovary of nude mice, and ES-2-HM cells were isolated from omental metastases after three cycles of in vivo selection. All cell lines were authenticated by short tandem repeat analysis within two years. All cells were cultured in DMEM/F12 (BasalMedia, China) supplemented with 10% fetal bovine serum (ExCell Bio, China) in a humidified atmosphere with 5% CO_2_ at 37 °C.

### Primary cell culture

Fresh omentum from 14 patients with a pathological diagnosis of OC without omental metastases (Supplementary Table 1) undergoing surgery in our hospital was used to isolate ADSCs within 2 hours after excision. ADSCs were isolated using the enzyme digestion method as described previously [8], and the 2nd-5th generations of ADSCs were used for this study. The cells were cultured in DMEM/F12 supplemented with 10% fetal bovine serum in a humidified atmosphere with 5% CO_2_ at 37 °C.

### Animal studies

Six-week-old female BALB/c nude mice were purchased from Beijing Vital River Laboratory Animal Co. for in vivo xenograft models. To explore the patterns of highly metastatic tumor cell sEV in shaping the metastatic niche, ovarian orthotopic xenografts were established with luciferase-labeled ES-2 cells as described previously [57]. Each mouse was injected intraperitoneally with PBS, 50 μg sEV^ES-2^ or sEV^ES-2-HM^ twice a week and dynamically monitored using an in vivo imaging system (LUMIN II, Caliper, USA). The nude mice were sacrificed when ascites or cachexia developed. To explore the role of miR-320a-activated ADSCs in OC metastasis, luciferase-labeled ES-2 orthotopic ovarian xenografts were injected intraperitoneally with PBS, ADSCs, ADSCs^NC^ or ADSCs^miR-320a^. The abdominal organs of the above mice were isolated for ex vivo imaging to detect the scope of tumor peritoneal dissemination. To investigate the effects of miR-320a-activated ADSCs on OC growth, SK-OV-3 cells were mixed with PBS, 2 × 10^5^ ADSCs, ADSCs^NC^ or ADSCs^miR-320a^ and injected subcutaneously into the right dorsal flank of each mouse. The formula for measuring the tumor volume is length × width^2^ × 0.5. Tumor foci and omentum of all the above mice were excised and formalin-fixed and paraffin-embedded for subsequent immunohistochemical and in situ hybridization staining.

### Tissue staining

Formalin-fixed and paraffin-embedded slides of ovarian tumor foci from 62 patients and omentum from 53 patients were used for immunohistochemical α-SMA staining and miR-320a staining by in situ hybridization as described previously [21]. The stained intensity (none = 0, weak = 1, moderate = 2, strong = 3) (Supplementary Fig. 11) multiplied by the stained area (none = 0, less than 30% = 1, between 30 and 60% = 2, more than 60% = 3) represents α-SMA and miR-320a expression.

### High-throughput miRNA sequencing of small extracellular vesicle

The miRNA transcriptional landscape between sEV^ES-2^ and sEV^ES-2-HM^ was detected using high-throughput sequencing on an Illumina HiSeqTM 2500 (RIBOBIO, Guangzhou, China). The differential miRNA profile was measured using the *t* test, and candidates with absolute values of logFC ≥ 1 and *P* < 0.05 were considered significant.

### High-throughput mRNA sequencing of ADSCs

To explore the potential downstream effector of miR-320a in ADSCs, the transcriptional landscape between ADSCs^NC^ and ADSCs^miR-320a^ was sequenced on an Illumina HiSeqTM 3000 (Seqhealth Technology Co LTD, Wuhan, China). The differentially expressed genes were identified using the DESeq2 package. A *P* value < 0.05 and an absolute value of logFC > 1 were set as the cutoff criteria.

### Statistical analysis

All statistical analyses were performed using GraphPad Prism 8.0 software. Numerical data are presented as the mean ± standard deviation. Differences between observation groups in the cell experiments were assessed using Student’s *t* test (two groups) and one-way ANOVA (three groups and more). The association between miR-320a and clinicopathological characteristics was evaluated using the chi-square test and Fisher’s exact test. The Kaplan–Meier method and log-rank test were used to assess the difference in overall survival between different groups. The Pearson method was used to analyze the correlation between two variates. *P* < 0.05 was considered statistically significant.

### Supplementary Information

**Additional file 1.**

**Additional file 2.**

**Additional file 3.**

## Data Availability

Public datasets of ovarian cancer analyzed in this study can be retrieved from the Gene Expression Omnibus and Kaplan–Meier Plotter website. Other data supporting the findings of this study are available upon reasonable request to the corresponding authors.

## Abbreviations

ADSC: adipose-derived mesenchymal stem cell
CAF: cancer-associated fibroblast
CM: conditioned medium
MSC: mesenchymal stem cell
OC: ovarian cancer
sEV: small extracellular vesicle
