# Supplementary material for: Tumor-derived small extracellular vesicles facilitate omental metastasis of ovarian cancer by triggering activation of mesenchymal stem cells
Source: Cell Commun Signal. 2024 Jan 17;22:47. doi: 10.1186/s12964-023-01413-9 (PMC10795335; doi:10.1186/s12964-023-01413-9)
Supplement: Supplementary file 2 — Additional file 1. [file 12964_2023_1413_MOESM1_ESM.pdf]

Detailed materials and methods

Isolation and identification of small extracellular vesicles

Ascites from twenty-two ovarian cancer (OC) patients (Supplementary Table 2) were used to isolate ascites-derived sEV. Briefly, fresh ascites and conditioned medium (CM) were centrifuged at 2000 x g for 10 min to remove large cell debris, 12 000 x g for 30 min to remove small cell debris and contaminants, and filtered with a 0.22 μm filter membrane (Millipore, USA). Subsequently, the supernatant was ultracentrifuged at 120 000 x g for 70 min with an Optima XPN-100 (Beckman Coulter, USA). Isolated small extracellular vesicles (sEV) were stored at -80°C for further study. The morphology of sEV was observed with a MEGAVIEW G2 transmission electron microscope (EMSIS Company, Germany), and the particle size distribution was assessed with a Nano series-Nano-ZS (ZETASIZER, UK). Furthermore, flow cytometry for sEV stained with FITC-labeled CD63 antibody and PE-labeled CD81 antibody was performed to detect the purity of isolated sEV.

Extracellular vesicle tracing assay

PKH67 (Sigma, USA) was used to stain sEV to detect the internalization of sEV by adipose-derived mesenchymal stem cells (ADSCs). Briefly, ADSCs were cultured in complete medium supplemented with 50 μg/mL PKH67-labeled sEV. After 24 hours of culture, the cells were fixed with 4% formaldehyde, stained with TRITC-labeled phalloidin, counterstained with DAPI, and photographed under a fluorescence microscope under 400x magnification.

Western blot

Western blot was performed to detect markers of sEV (ALIX, CD63, CD9, CD81 and

Calnexin), fibroblast activation markers (α-SMA and FAP), the PI3K-AKT pathway (AKT and phosphorylated AKT), the ERK-MAPK pathway (ERK1 and phosphorylated ERK1), and the TGF-beta pathway (TGF-β1, SMAD2, phosphorylated SMAD2, SMAD3 and phosphorylated SMAD3). Briefly, total proteins of cells and sEV were homogenized with NP40 lysis buffer and quantified with the bicinchoninic acid (P0011, Beyotime, China) method. The proteins were loaded onto 10% SDS–PAGE gels at 10 μg per lane for electrophoresis and then transferred to PVDF membranes. After blocking with 5% skim milk for two hours, the membrane was incubated with specific primary antibodies (Supplementary Table 3) overnight at 4°C. The next day, the membrane was incubated with HRP-labeled secondary antibodies followed by visualization with ECL chemiluminescent substrate (BL523A, Biosharp, China) in a Chemiluminescence Imager (Bio-Rad, USA). Each assay was performed with three biological replicates.

Real-time quantitative PCR (RT–qPCR)

Total RNA of cells and sEV were isolated using an RNA isolater (Vazyme, China). Reverse transcription was performed using the HiScript II qRT SuperMix Kit with gDNA Eraser (Enzyme, China) and PrimeScript^{TM} RT Reagent Kit (Takara, Japan) for mRNA and miRNA, respectively. qPCRs were performed using AceQ qPCR SYBR Green Master Mix (Vazyme, China) in an ABI 7500/7500 Fast Real-Time PCR System (Applied Biosystems, CA, USA). *ACTB* and *U6* were used as the internal controls for mRNA and miRNA, respectively. All miRNA and *U6* primers were purchased from RIBOBIO (Guangzhou, China), and the mRNA primers were synthesized by Tsingke Biotech Co. (Wuhan, China). The sequences of all primers are listed in Supplementary Table 4. Each assay was performed in triplicate with at least three biological replicates.

Immunofluorescence

The immunofluorescence assay was performed to visualize cellular α-SMA levels and localization. Briefly, 5×10^4 ADSCs per well were grown in 12-well plates and fixed with 4% paraformaldehyde. Subsequently, the cells were incubated with 0.5% Triton X-100 to break the cytomembrane, followed by incubation with anti-α-SMA antibody overnight at 4°C. The cells were incubated with Cy3-labeled fluorescent secondary antibody for two hours the next day, followed by DAPI (Boster, China) staining. Representative fields were photographed under a fluorescence microscope.

Migration and invasion assay

To detect the migratory and invasive abilities of cells *in vitro*, OC cells (4×10^4 cells per well) or ADSCs (1×10^4 cells per well) were suspended in 200 μL DMEM/F12 and cultured in the upper chambers of a Transwell plate (Corning, USA), and the lower chambers were filled with complete medium. After culture, the cells that passed through the membrane were fixed with 4% paraformaldehyde and stained with 0.1% crystal violet, followed by photographs under 100x magnification. The migratory and invasive properties were assessed by the number of cells that passed through the membrane in five random images. Each assay was performed with at least three biological replicates.

Wound-healing assay

Equivalent numbers of OC cells were grown in a 6-well plate to form a confluent monolayer and scratched with a 200 μL pipette tip to form a wound. The wound was photographed when scratched and 24 hours later, and the healing area was calculated using ImageJ software. The healing area represents the migrative properties of OC cells.

Each assay was performed with at least three biological replicates.

EdU proliferation assays

An EdU assay was used to detect cellular proliferation (RIBOBIO, Guangzhou, China). The percentage of EdU-positive cells and Hoechst-positive cells was used to assess the proliferative properties of cells. Each assay was performed with three biological replicates.

Colony formation assay

A total of 500 cells per well were grown in 6-well plates until visible colony formation. The cells were fixed with 4% paraformaldehyde and stained with 0.1% crystal violet. The number of colonies was counted using ImageJ software and represents the proliferative capacity of cells. Each assay was performed with three biological replicates.

Cell viability assay

Cell viability was detected using a Cell Counting Kit (DOJINDO, Japan). Briefly, 8000 cells per well were grown in 96-well plates for 24, 48 and 72 hours. After four hours of incubation with CCK8 solution, the absorbance of the incubation solution at 450 nm was detected by a microplate reader (SpectraMax i3x, USA). Each assay was performed with three biological replicates.

Expression modulation

The miRNA mimics and inhibitors of miR-24-3p, miR-320a, miR-320d, miR-27-3p and miR-320c and small interfering RNAs of *RCN2*, *PDIA2*, *ITGA7*, *ABCG1*, *UROS*

and *GAL3ST2* were purchased from RIBOBIO (Guangzhou, China) for modulation of the cellular transcriptome. The transfection of tumor cells and ADSCs was performed using Lipofectamine 2000 (Invitrogen, USA) and HiPerFect (Qiagen, USA), respectively.

Dual-luciferase reporter assay

The wild-type or mutated sequence of the *ITGA7* or *UROS* mRNA 3'UTR was cloned into the Renilla luciferase plasmid. $ADSCs^{NC}$ and $ADSCs^{miR\text{-}320a}$ were transfected with specific luciferase reporter plasmids. After 48 hours of culture, the bioluminescence signal was detected using a Dual-Glo luciferase assay kit (Promega, E2920, USA) in a GloMax20/20 luminometer. Each assay was performed with three biological replicates.

Bioinformatic analysis

The high-quality OC miRNA array dataset GSE73581 deposited in Gene Expression Omnibus was used to investigate the relationship between miR-320a expression and overall survival and time to relapse of patients. The correlation between *ITGA7* and overall survival and progression-free survival in patients with advanced OC was assessed on the Kaplan–Meier Plotter website (http://kmplot.com), retrieved on June 15, 2023. The criteria are stage III/IV OC patients who have undergone optimal debulking surgery.
