# Supplementary material for: Tumor-derived small extracellular vesicles facilitate omental metastasis of ovarian cancer by triggering activation of mesenchymal stem cells
Source: Cell Commun Signal. 2024 Jan 17;22:47. doi: 10.1186/s12964-023-01413-9 (PMC10795335; doi:10.1186/s12964-023-01413-9)
Supplement: Supplementary file 3 — Additional file 2. [file 12964_2023_1413_MOESM2_ESM.pdf]

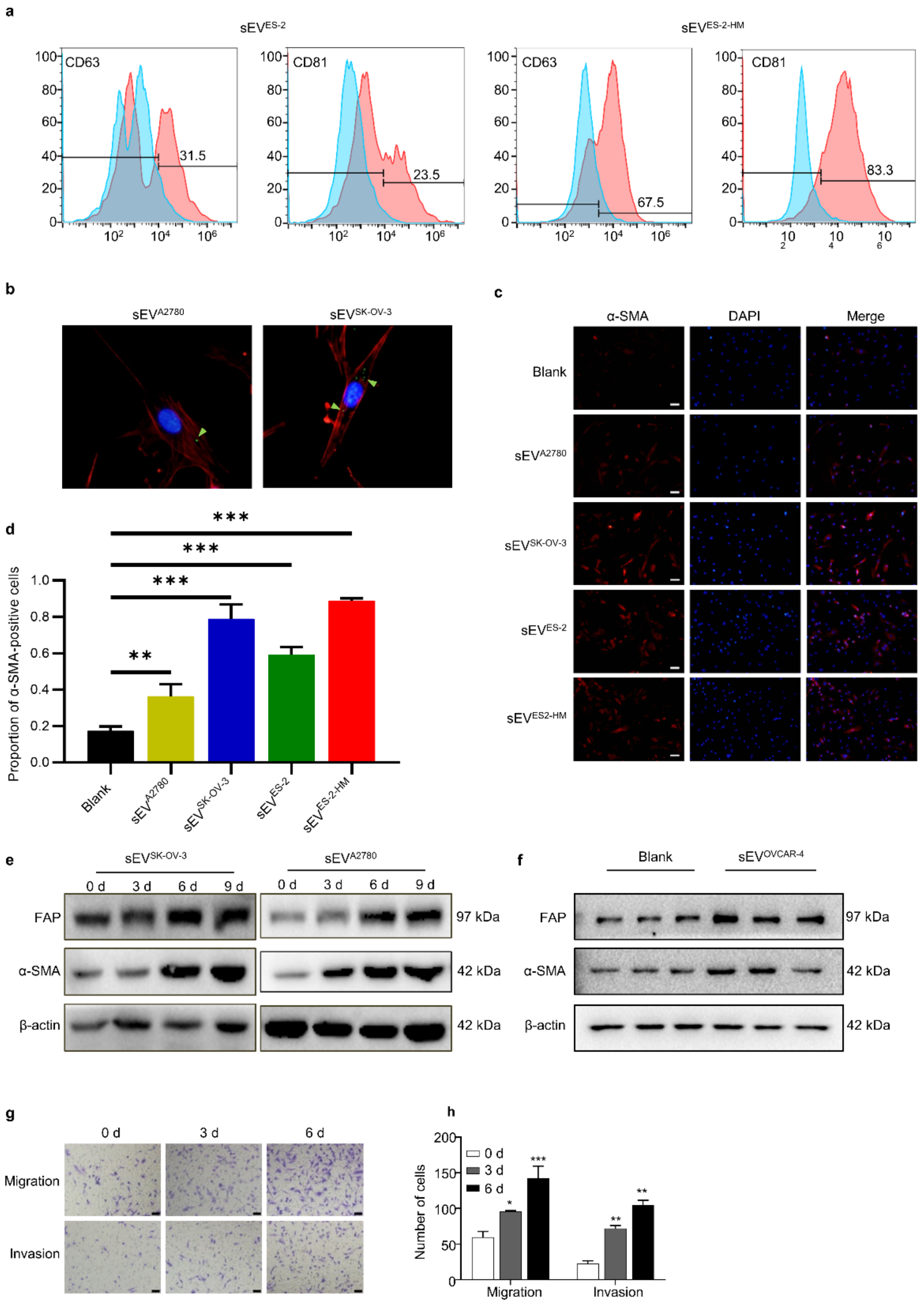

Supplementary Fig. 1 Small extracellular vesicles derived from ovarian cancer cells induce activation of ADSCs toward CAFs. (a) Flow cytometry analysis for CD63 and CD9 on small extracellular vesicles (sEV) derived from ES-2 (sEV$^{\text{ES-2}}$) and ES-2-HM cells (sEV$^{\text{ES-2-HM}}$). (b) Representative images of adipose-derived mesenchymal stem cells (ADSCs) uptake of PKH67-labeled sEV derived from A2780 (sEV$^{\text{A2780}}$) and SK-OV-3 cells (sEV$^{\text{SK-OV-3}}$). Red staining represents the cytoskeleton, green staining represents the sEV and blue staining represents the nucleus. (c) The levels and localization for α-SMA of ADSCs cocultured with sEV$^{\text{A2780}}$, sEV$^{\text{SK-OV-3}}$, sEV$^{\text{ES-2}}$ or sEV$^{\text{ES-2-HM}}$ examined by immunofluorescence. Scale bar, 200 μm. (d) Quantification of α-SMA immunofluorescence in ADSCs cocultured with sEV$^{\text{A2780}}$, sEV$^{\text{SK-OV-3}}$,

sEV^{ES-2} or $sEV^{ES-2-HM}$. (e) FAP and α-SMA levels of ADSCs cocultured with sEV^{A2780} and $sEV^{SK-OV-3}$ for different durations (0, 3, 6 and 9 days) examined by Western blot. (f) FAP and α-SMA levels of ADSCs cocultured with sEV derived from OVCAR-4 cells examined by Western blot. (g) Representative images and (h) statistical results for migration and invasion assays demonstrate the migratory and invasive abilities of ADSCs cocultured with ascites-derived sEV of ovarian cancer patients for different durations (0, 3 and 6 days). Scale bar, 50 μm. * $P < 0.05$, ** $P < 0.01$, *** $P < 0.001$.

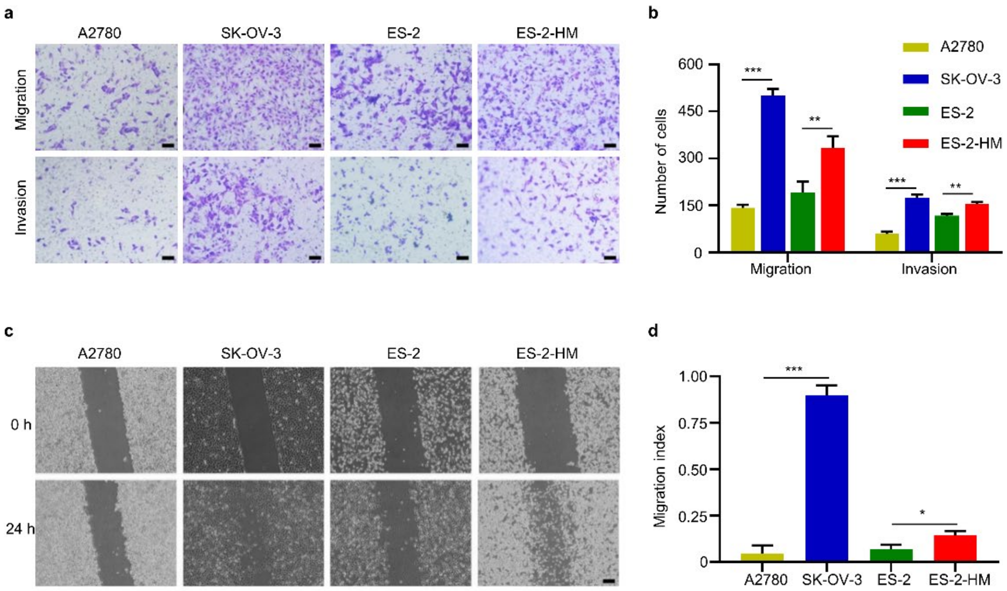

Supplementary Fig. 2 Migrative and invasive capacity of ovarian cancer cells. (a) Representative images of migration and invasion assays demonstrate the migratory and invasive abilities of four ovarian cancer cell lines (A2780, SK-OV-3, ES-2, and ES-2-HM). Scale bar, 100 μm. (b) Statistical results of migration, invasion of A2780, SK-OV-3, ES-2, and ES-2-HM cells. (c) Representative images of Wound healing assay demonstrate the motility of A2780, SK-OV-3, ES-2, and ES-2-HM cells. Scale bar, 200 μm. (d) Statistical results of Wound healing assay of A2780, SK-OV-3, ES-2, and ES-2-HM cells. * $P < 0.05$, ** $P < 0.01$, *** $P < 0.001$.

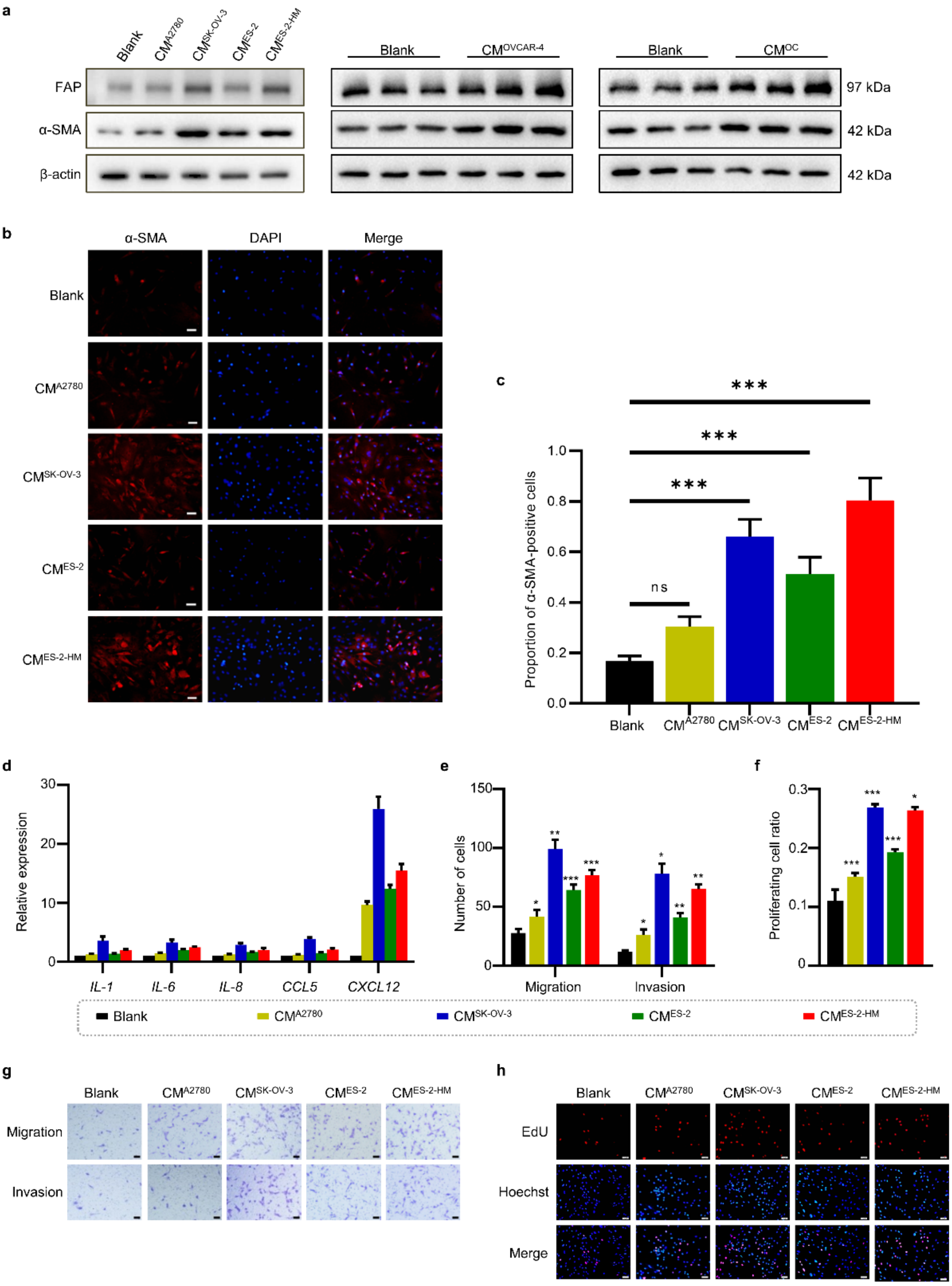

Supplementary Fig. 3 Ovarian cancer induced the activation of ADSCs toward CAFs. (a) FAP and α-SMA levels of ADSCs cocultured with conditioned medium (CM) of A2780 (CM^{A2780}), SK-OV-3 ($CM^{SK\text{-}OV\text{-}3}$), ES-2($CM^{ES\text{-}2}$), ES-2-HM cells ($CM^{ES\text{-}2\text{-}HM}$), OVCAR-4 ($CM^{OVCAR\text{-}4}$) or primary ovarian cancer (CM^{OC}) cells examined by Western blot. (b) The levels and localization of α-SMA of ADSCs cocultured with CM^{A2780}, $CM^{SK\text{-}OV\text{-}3}$, $CM^{ES\text{-}2}$ or $CM^{ES\text{-}2\text{-}HM}$ examined by immunofluorescence. Scale bar, 200 μm. (c) Quantification of α-SMA immunofluorescence in ADSCs cocultured with CM^{A2780}, $CM^{SK\text{-}OV\text{-}3}$, $CM^{ES\text{-}2}$ or $CM^{ES\text{-}2\text{-}HM}$. (d) Expression of *IL-1*, *IL-6*, *IL-8*, *CCL5* and *CXCL12* in ADSCs cocultured with CM^{A2780}, $CM^{SK\text{-}OV\text{-}3}$, $CM^{ES\text{-}2}$ or $CM^{ES\text{-}2\text{-}HM}$ detected by RT–qPCR. (e) Statistical results of migration and invasion of ADSCs cocultured with CM^{A2780}, $CM^{SK\text{-}OV\text{-}3}$, $CM^{ES\text{-}2}$ or $CM^{ES\text{-}2\text{-}HM}$. (f) Statistical results show alterations in the

proliferation capacity of ADSCs cocultured with CM^{A2780}, $CM^{SK\text{-}OV\text{-}3}$, $CM^{ES\text{-}2}$ or $CM^{ES\text{-}2\text{-}HM}$. (g) Representative images of migration and invasion assays demonstrate the migratory and invasive abilities of ADSCs cocultured with CM^{A2780}, $CM^{SK\text{-}OV\text{-}3}$, $CM^{ES\text{-}2}$ or $CM^{ES\text{-}2\text{-}HM}$. Scale bar, 50 μm. (h) Representative images of the EdU assay demonstrate the proliferative capacity of ADSCs cocultured with CM^{A2780}, $CM^{SK\text{-}OV\text{-}3}$, $CM^{ES\text{-}2}$ or $CM^{ES\text{-}2\text{-}HM}$. Scale bar, 50 μm. * $P < 0.05$, ** $P < 0.01$, *** $P < 0.001$.

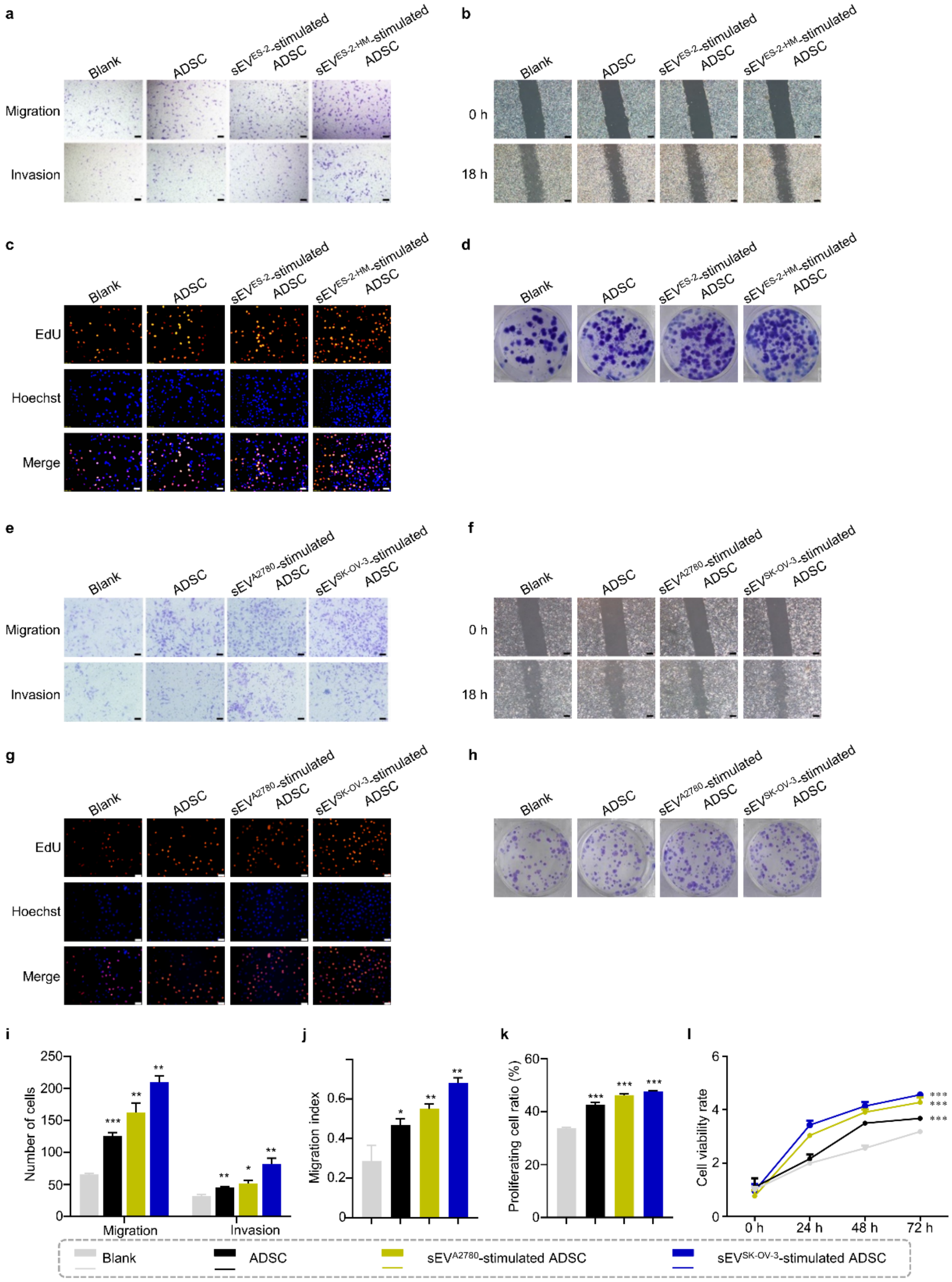

Supplementary Fig. 4 Small extracellular vesicle-activated ADSCs promote ovarian cancer cell proliferation, migration and invasion *in vitro*. (a) Representative images of the migration and invasion assays demonstrate the migratory and invasive abilities of ES-2 cells cocultured with ADSCs, sEV^{ES-2}-stimulated ADSCs or sEV$^{ES-2-HM}$-stimulated ADSCs. Scale bar, 50 μm. (b) Representative images of the Wound healing assay demonstrate the motility of ES-2 cells cocultured with ADSCs, sEV^{ES-2}-stimulated ADSCs or sEV$^{ES-2-HM}$-stimulated ADSCs. Scale bar, 200 μm. (c) Representative images of the EdU assay demonstrate the proliferative capacity of ES-2 cells cocultured with ADSCs, sEV^{ES-2}-stimulated ADSCs or sEV$^{ES-2-HM}$-stimulated ADSCs. Scale bar, 50 μm. (d) Representative images of the colony formation assay of ES-2 cells

cocultured with ADSCs, sEV^{ES-2}-stimulated ADSCs or sEV$^{ES-2-HM}$-stimulated ADSCs. (e) Representative images of the migration and invasion assays demonstrate the migratory and invasive abilities of SK-OV-3 cells cocultured with ADSCs, sEV^{A2780}-stimulated ADSCs or sEV$^{SK-OV-3}$-stimulated ADSCs. Scale bar, 50 μm. (f) Representative images of the Wound healing assay demonstrate the motility of SK-OV-3 cells cocultured with ADSCs, sEV^{A2780}-stimulated ADSCs or sEV$^{SK-OV-3}$-stimulated ADSCs. Scale bar, 200 μm. (g) Representative images of the EdU assay demonstrate the proliferative capacity of SK-OV-3 cells cocultured with ADSCs, sEV^{A2780}-stimulated ADSCs or sEV$^{SK-OV-3}$-stimulated ADSCs. Scale bar, 50 μm. (h) Representative images of the colony formation assay of SK-OV-3 cells cocultured with ADSCs, sEV^{A2780}-stimulated ADSCs or sEV$^{SK-OV-3}$-stimulated ADSCs. (i) Statistical results of migration, invasion of SK-OV-3 cells cocultured with ADSCs, sEV^{A2780}-stimulated ADSCs or sEV$^{SK-OV-3}$-stimulated ADSCs. (j) Statistical results of the Wound healing assay demonstrate the motility of SK-OV-3 cells cocultured with ADSCs, sEV^{A2780}-stimulated ADSCs or sEV$^{SK-OV-3}$-stimulated ADSCs. (k) Statistical results showing alterations in the proliferation capacity of SK-OV-3 cells cocultured with ADSCs, sEV^{A2780}-stimulated ADSCs or sEV$^{SK-OV-3}$-stimulated ADSCs. (l) Statistical results of the viability of SK-OV-3 cells cocultured with ADSCs, sEV^{A2780}-stimulated ADSCs or sEV$^{SK-OV-3}$-stimulated ADSCs. * $P < 0.05$, ** $P < 0.01$, *** $P < 0.001$.

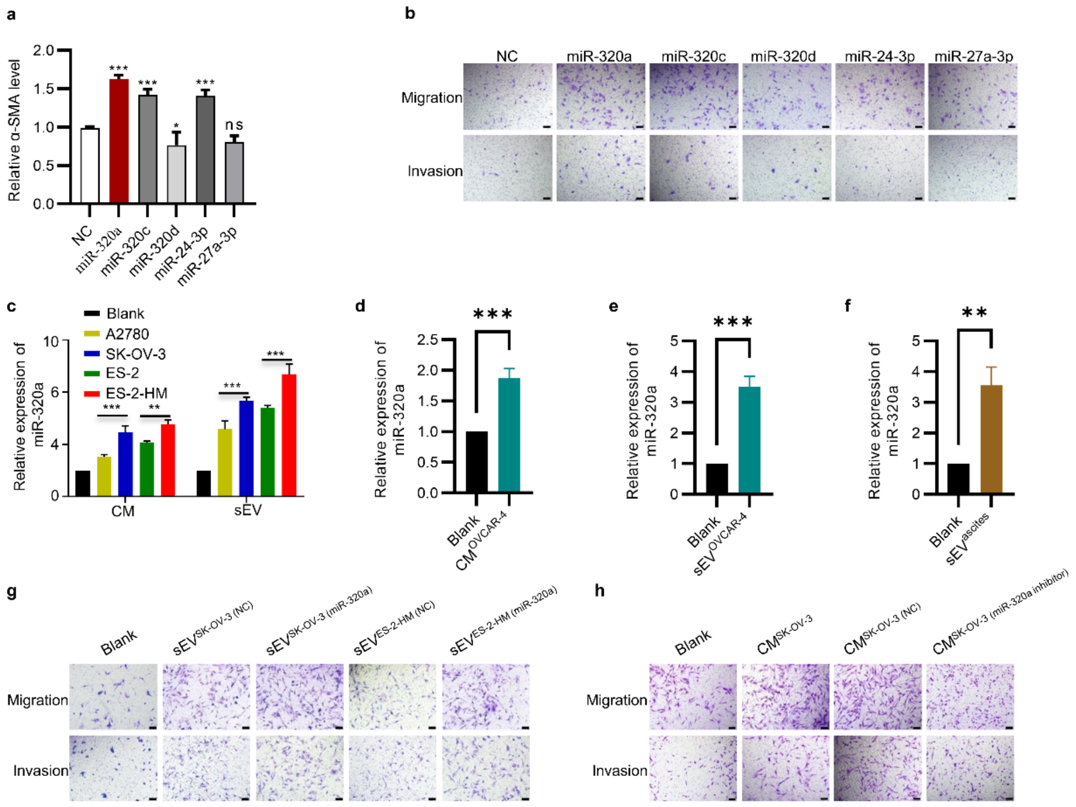

Supplementary Fig. 5 miR-320a in small extracellular vesicles enhances the motility and invasiveness of ADSCs *in vitro*. (a) Quantification of α-SMA levels in ADSCs overexpressing miR-320a, miR-320c, miR-320d, miR-24-3p, or miR-27a-3p, as determined by Western blot. (b) Representative images of the migration and invasion assays demonstrate the migratory and invasive abilities of ADSCs overexpressing miR-320a, miR-320c, miR-320d, miR-24-3p or miR-27a-3p. Scale bar, 50 μm. (c) Relative expression of miR-320a of ADSCs coincubated with conditioned medium (CM) or small extracellular vesicles (sEV) derived from A2780, SK-OV-3, ES-2 or ES-2-HM cells detected by RT–qPCR. (d) Relative expression of miR-320a of ADSCs coincubated with CM derived from OVCAR-4 cells detected by RT–qPCR. (e) Relative expression of miR-320a of ADSCs coincubated with sEV derived from OVCAR-4 cells detected by RT–qPCR. (f) Relative expression of miR-320a of ADSCs coincubated with sEV derived from ascites of high-grade serous ovarian cancer patient detected by RT–qPCR. (g) Representative images of the migration and invasion assays demonstrate the migratory and invasive abilities of ADSCs cocultured with sEV derived from SK-OV-3 negative control ($sEV^{SK\text{-}OV\text{-}3\ (NC)}$), SK-OV-3 overexpressing miR-320a ($sEV^{SK\text{-}OV\text{-}3\ (miR\text{-}320a)}$), ES-2-HM negative control ($sEV^{ES\text{-}2\text{-}HM\ (NC)}$) or ES-2-HM overexpressing miR-320a ($sEV^{ES\text{-}2\text{-}HM\ (miR\text{-}320a)}$). Scale bar, 50 μm. (h) Representative images of the migration and invasion assays demonstrate the migratory and invasive abilities of ADSCs cocultured with CM derived from SK-OV-3, SK-OV-3 negative control ($CM^{SK\text{-}OV\text{-}3\ (NC)}$), or SK-OV-3 with miR-320a inhibition ($CM^{SK\text{-}OV\text{-}3\ (miR\text{-}320a\ inhibitor)}$). Scale bar, 50 μm. (d) ** $P < 0.01$, *** $P < 0.001$.

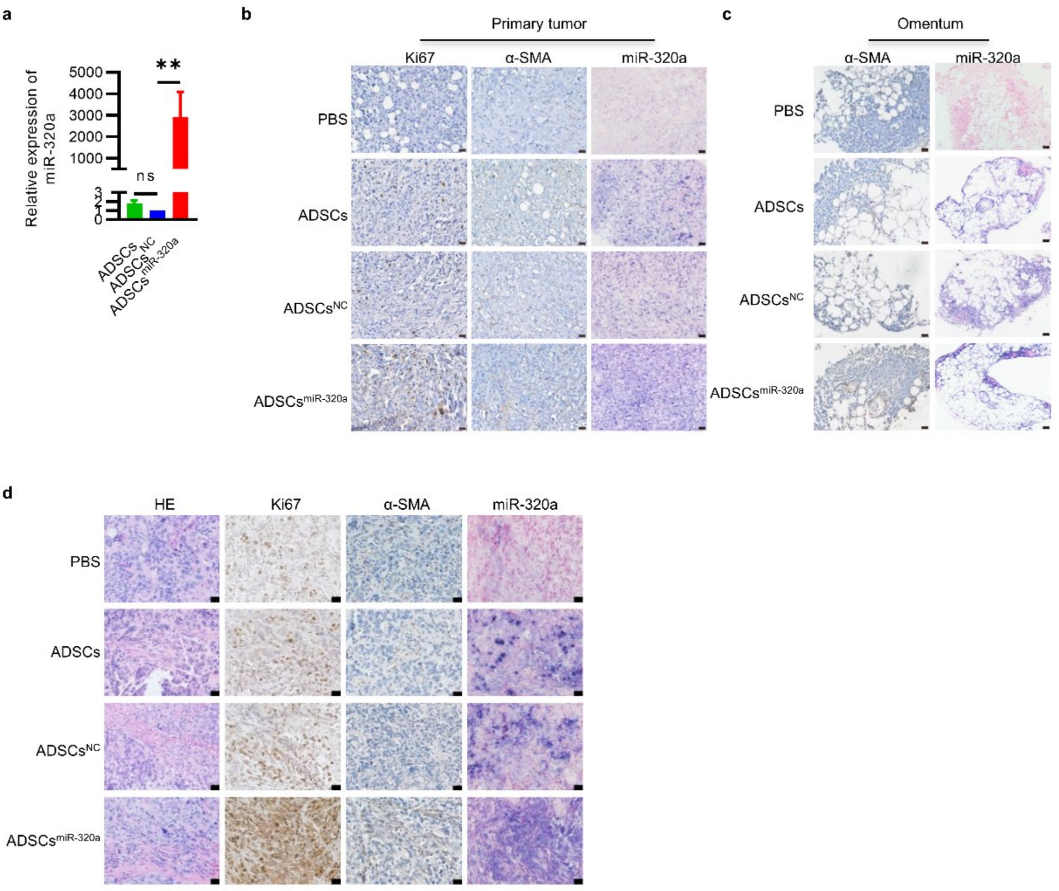

Supplementary Fig. 6 miR-320a-activated ADSCs promote activation of the omental metastatic microenvironment. (a) Relative expression of miR-320a in ADSCs, which were injected into mice bearing ovarian orthotopic engraftment tumors and subcutaneous xenograft tumors. (b) Representative images of miR-320a, α-SMA and Ki67 staining in tumor foci of ovarian orthotopic engraftment tumor-bearing mice injected intraperitoneally with PBS, ADSCs, negative control ADSCs (ADSCs^{NC}) or ADSCs overexpressing miR-320a (ADSCs$^{miR\text{-}320a}$). Scale bar, 20 μm. (c) Representative images of miR-320a and α-SMA staining in the omentum of the above mice. Scale bar, 20 μm. (d) Representative images for miR-320a, α-SMA and Ki67 staining of SK-OV-3 subcutaneous xenograft tumors mixed with PBS, ADSCs, ADSCs^{NC} or ADSCs$^{miR\text{-}320a}$. ns, no significance, ** $P < 0.01$.

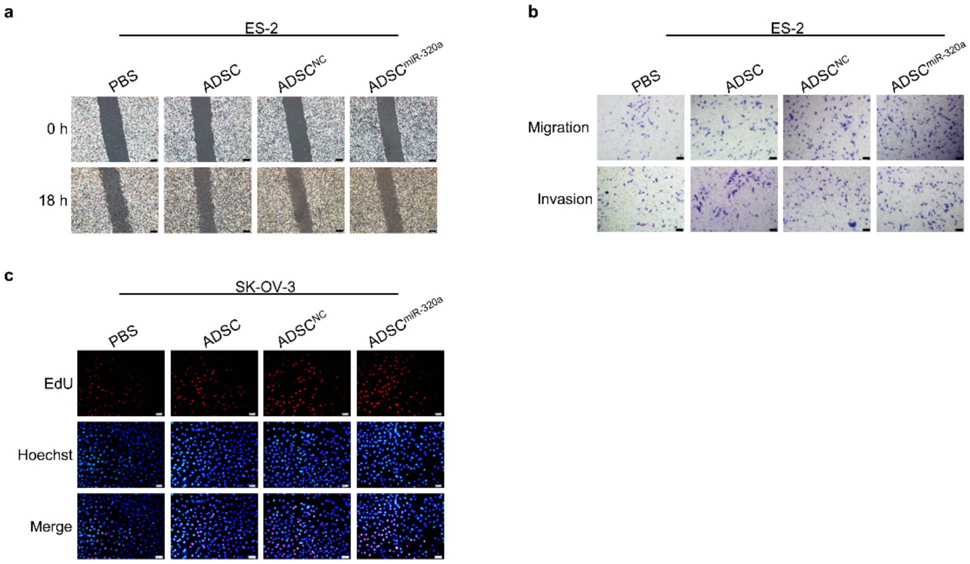

Supplementary Fig. 7 miR-320a-activated ADSCs enhance the motility and invasiveness of ovarian cancer cells. (a) Representative images of the Wound healing assay demonstrate the motility of ES-2 cells cocultured with conditioned medium derived from ADSCs, ADSCs negative control (ADSCs^{NC}), or ADSCs overexpressing miR-320a (ADSCs$^{miR\text{-}320a}$). Scale bar, 200 µm. (b) Representative images of the migration and invasion assays demonstrate the migratory and invasive abilities of ES-2 cells indirectly cocultured with ADSCs, ADSCs^{NC}, or ADSCs$^{miR\text{-}320a}$. Scale bar, 50 µm. (c) Representative images of the EdU assay demonstrate the proliferative capacity of SK-OV-3 cells indirectly cocultured with ADSCs, ADSCs^{NC}, or ADSCs$^{miR\text{-}320a}$. Scale bar, 50 µm.

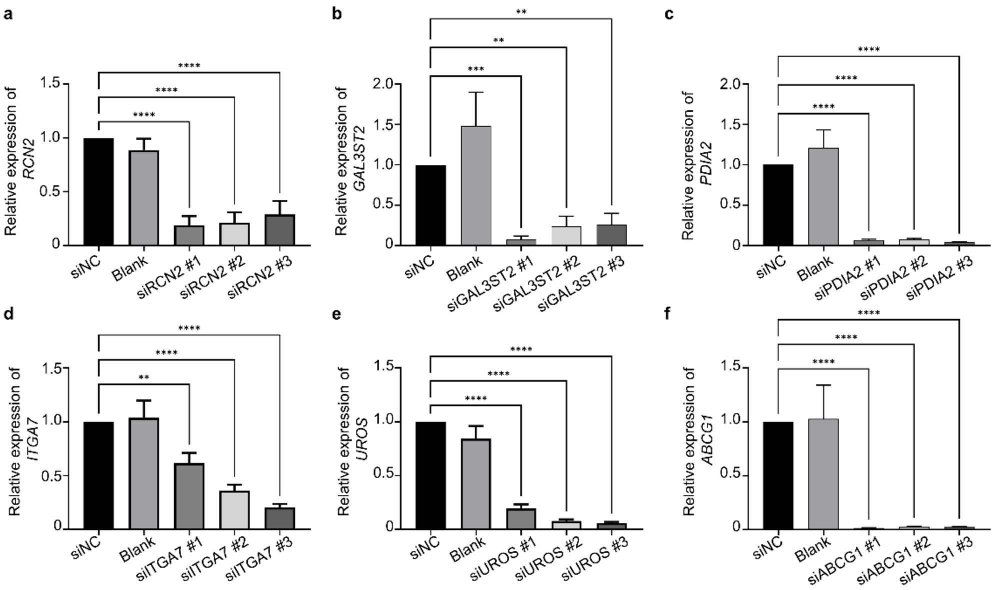

Supplementary Fig. 8 Detection of interfering efficiency of siRNAs targeting *RCN2*, *GAL3ST2*, *PDIA2*, *ITGA7*, *UROS*, and *ABCG1*. Relative expression of *RCN2* (a), *GAL3ST2* (b), *PDIA2* (c), *ITGA7* (d), *UROS* (e), and *ABCG1* (f) in ADSCs after transfection with the corresponding small interfering RNA detected by RT–qPCR. * $P < 0.05$, ** $P < 0.01$, *** $P < 0.001$, **** $P < 0.0001$.

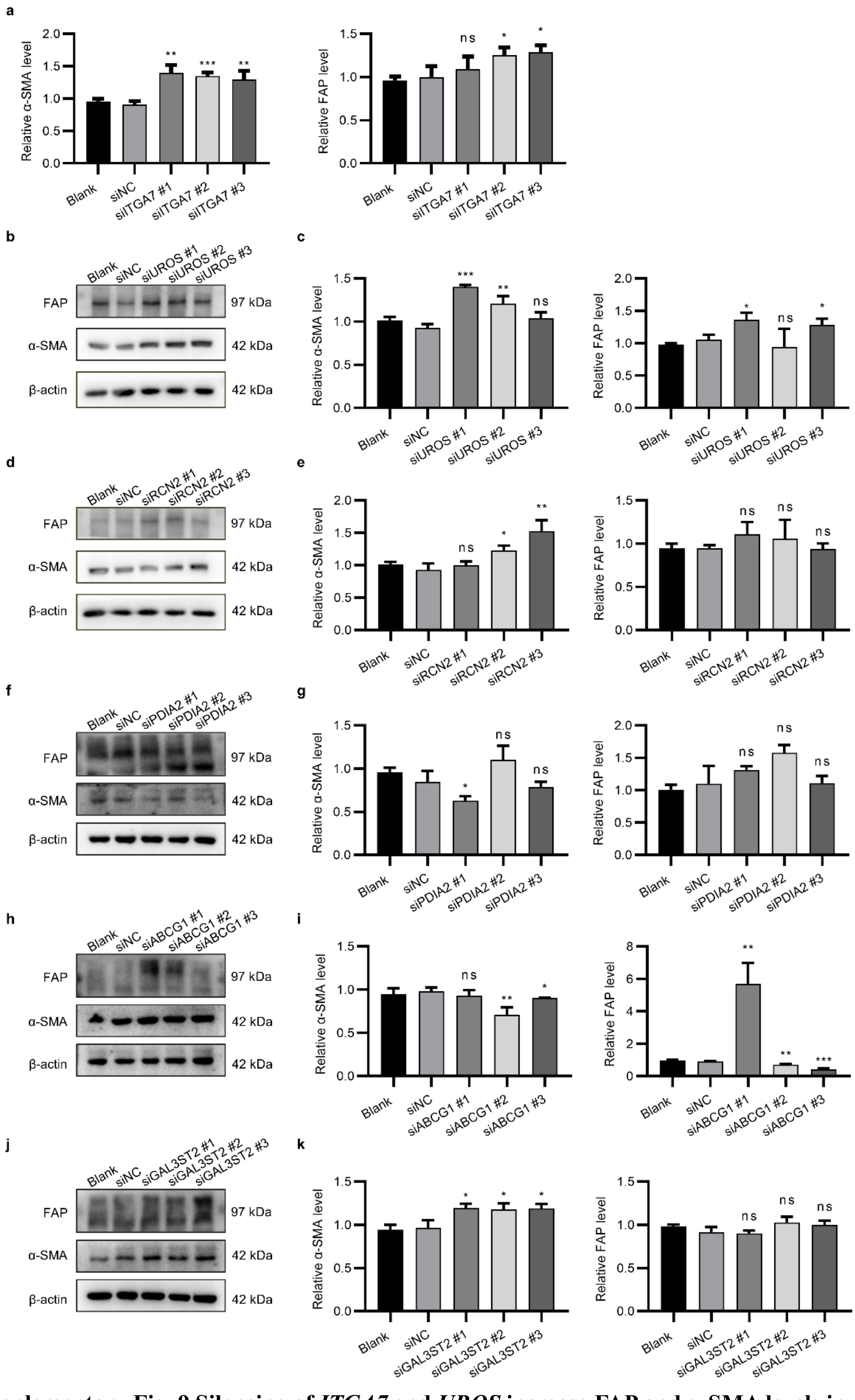

Supplementary Fig. 9 Silencing of *ITGA7* and *UROS* increase FAP and α-SMA levels in ADSCs. (a) Quantification of α-SMA (left) and FAP (right) levels in ADSCs silencing *ITGA7*, as determined by Western blot. (b) Representative Western blot images of α-SMA and FAP levels of ADSCs with silencing for *UROS* (c) Quantification of α-SMA (left) and FAP (right) levels in ADSCs silencing *UROS*, as determined by Western blot. (d) Representative Western blot images

of α-SMA and FAP levels of ADSCs with silencing for *RCN2*. (e) Quantification of α-SMA (left) and FAP (right) levels in ADSCs silencing *RCN2*, as determined by Western blot. (f) Representative Western blot images of α-SMA and FAP levels of ADSCs with silencing for *PDIA2*. (g) Quantification of α-SMA (left) and FAP (right) levels in ADSCs silencing *PDIA2*, as determined by Western blot. (h) Representative Western blot images of α-SMA and FAP levels of ADSCs with silencing for *ABCG1*. (i) Quantification of α-SMA (left) and FAP (right) levels in ADSCs silencing *ABCG1*, as determined by Western blot. (j) Representative Western blot images of α-SMA and FAP levels of ADSCs with silencing for *GAL3ST2*. (k) Quantification of α-SMA (left) and FAP (right) levels in ADSCs silencing *GAL3ST2*, as determined by Western blot. ns, no significance, * $P < 0.05$, ** $P < 0.01$, *** $P < 0.001$.

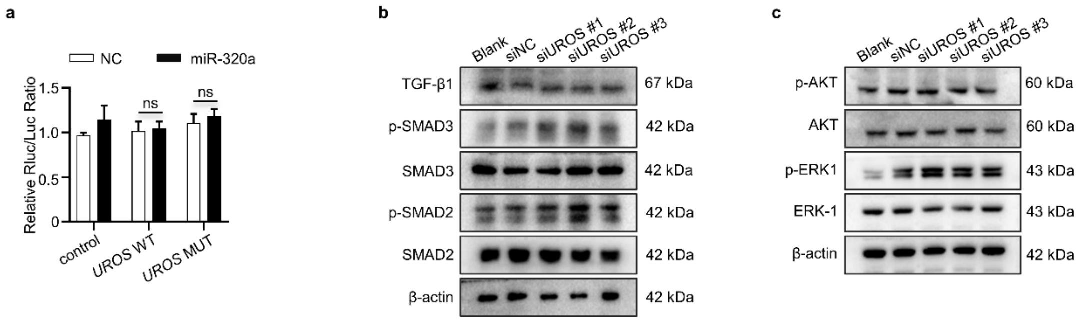

Supplementary Fig. 10 Silencing of *UROS* activated the ERK-MAPK pathways without affecting the TGF-β pathway. (a) Luciferase activity in ADSCs transfected with Renilla luciferase plasmid with *UROS* mutation detected by dual luciferase assay. (b) Levels of TGF-β1, SMAD2, SMAD3, phosphorylated SMAD2 (p-SMAD2) and phosphorylated SMAD3 (p-SMAD3) of *UROS*-silenced ADSCs detected by Western blot. (c) Levels of AKT, ERK1, phosphorylated AKT (p-AKT) and phosphorylated ERK1 (p-ERK1) of *UROS*-silenced ADSCs detected by Western blot. ns, no significance.

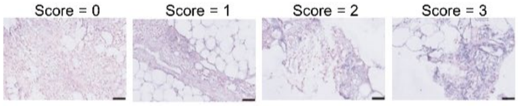

Supplementary Fig. 11 The expression of miR-320a in ovarian cancer. The score of miR-320a staining intensity in ovarian cancer.
