# Supplementary material for: Tumor-derived small extracellular vesicles facilitate omental metastasis of ovarian cancer by triggering activation of mesenchymal stem cells
Source: Cell Commun Signal. 2024 Jan 17;22:47. doi: 10.1186/s12964-023-01413-9 (PMC10795335; doi:10.1186/s12964-023-01413-9)
Supplement: Supplementary file 4 — Additional file 3. [file 12964_2023_1413_MOESM3_ESM.pdf]

Supplementary Table 1 The clinicopathological features of fourteen ovarian cancer patients for ADSC isolation.

ID	Age (years)	Pathological type	FIGO stage
#1	55	Endometrial adenocarcinoma	IA
#2	45	Adult granulosa cell tumor	IA
#3	63	Adult granulosa cell tumor	IA
#4	75	Clear cell carcinoma	IA
#5	58	Mucinous adenocarcinoma	IA
#6	55	Clear cell carcinoma	IA
#7	45	Mucinous carcinoma	IC
#8	56	Mucinous carcinoma	IA
#9	63	Borderline mucinous neoplasm	IC
#10	57	Clear cell carcinoma	IA
#11	45	Clear cell carcinoma	IA
#12	25	Borderline carcinoma	IA
#13	49	Borderline mucinous neoplasm	IA
#14	37	Borderline mucinous neoplasm	IA

ADSC, adipose-derived mesenchymal stem cell; FIGO, International Federation of Gynecology and Obstetrics.

Supplementary Table 2 The clinicopathological features of twenty-two ovarian cancer patients whose ascites was used to isolate exosomes

Clinicopathological features	*n* (%)
**Age (Years)**	
≤ 49	8 (36.36)
> 49	14 (63.64)
**Pathological types**	
HGSOC	20 (90.91)
Non-HGSOC	2 (9.09)
**FIGO stage**	
I – II	1 (4.55)
III – IV	21 (95.45)
**Omentum metastasis**	
Absent	4 (18.18)
Present	18 (81.82)

FIGO, International Federation of Gynecology and Obstetrics; HGSOC, high-grade serous ovarian cancer

Supplementary Table 3 The antibodies used in this study

Antibodies	Dilution	Vendor	Item No.	Nation	Application
CD9	1:1000	Abcam	ab236630	USA	Western blot
CD63	1:1000	Abcam	ab134045	USA	Western blot
CD81	1:1000	Abcam	ab109201	USA	Western blot
ALIX	1:1000	Abcam	ab186728	USA	Western blot
Calnexin	1:2000	Abcam	ab133615	USA	Western blot
α-SMA	1:5000	Abcam	ab5694	USA	Western blot
FAP	1:1000	R&D Systems	AF3715-SP	USA	Western blot
ITGA7	1:1000	Abcam	ab75224	USA	Western blot
TGF-β1	1:1000	Abcam	ab92486	USA	Western blot
SMAD2	1:2000	Cell Signaling Technology	3103	USA	Western blot
p-SMAD2	1:2000	Cell Signaling Technology	18338	USA	Western blot
SMAD3	1:2000	Cell Signaling Technology	9513	USA	Western blot
p-SMAD3	1:2000	Cell Signaling Technology	9520	USA	Western blot
AKT	1:2000	Cell Signaling Technology	2920	USA	Western blot
p-AKT	1:2000	Cell Signaling Technology	9611	USA	Western blot
MAPK	1:2000	Cell Signaling Technology	4695	USA	Western blot
p-MAPK	1:2000	Cell Signaling Technology	4370	USA	Western blot
GAPDH	1:5000	Proteintech	60004-1-Ig	China	Western blot
β-actin	1:5000	Proteintech	66009-1-Ig	China	Western blot
α-SMA	1:500	Abcam	ab5694	USA	Immunohistochemistry
Ki67	1:100	Affinity Biosciences	AF-0198	USA	Immunohistochemistry
ITGA7	1:100	Abcam	ab75224	USA	Immunohistochemistry
α-SMA	1:200	Abcam	ab5694	USA	Immunofluorescence
Cy3-labeled fluorescent antibody	1:5000	Proteintech	SA00009-2	China	Immunofluorescence
FITC-labeled CD63	-	BD Biosciences	557288	USA	Flow cytometry
PE-labeled CD81	-	BD Biosciences	555676	USA	Flow cytometry

Supplementary Table 4 The primer sequences used in this study

Gene names	Forward primer (5' ~ 3')	Reverse primer (5' ~ 3')
ACTB	GCCAACACAGTGCTGTCTGG	GCTCAGGAGGAGCAATGATCTTG
IL-1	ATGATGGCTTATTACAGTGGCAA	GTCGGAGATTCGTAGCTGGA
IL-6	ACTCACCTCTTCAGAACGAATTG	CCATCTTTGGAAGGTTCAGGTTG
IL-8	ACTGAGAGTGATTGAGAGTGGAC	AACCCTCTGCACCCAGTTTTC
CCL5	CCAGCAGTCGTCTTTGTCAC	CTCTGGGTTGGCACACACTT
CXCL12	ATTCTCAACACTCCAAACTGTGC	ACTTTAGCTTCGGGTCAATGC
MEST	ATCGGGTGATTGCCCTTGATT	GAAAGAAGGTTGATCCTGCGG
HSD3B1	CACATGGCCCGCTCCATAC	GTGCCGCCGTTTTTCAGATTC
STABI	CACATGGCCCGCTCCATAC	GTGCCGCCGTTTTTCAGATTC
AGFG1	TCTCTTTTAGGGGATTCTGCACC	AGAACGACCTACAACTGGGGA
ARHGAP28	CGGTTCCCAGGAGTGACTCT	CAGGCTCCGGTGATCCATTG
CLDN22	GAGGGCGATCTTGGGTTTTG	ATATGTGCTCTGTGGTCGCT
FGF9	GGCCTGGTCAGCATTCGAG	GTATCGCCTTCCAGTGTCCAC
NRXN3	CAGCACGAGGATGGATCGC	GCCCACGTTAAAGATCACCCC
RCN2	TGGACTCAGATGGCTTTCTCA	GACCTGAATCCTGGTTAGCTTTT
PDIA2	GCTGAGGAGTTTGGTGTGAC	AGAAGCCAATGACCACTAGGT
ITGA7	CTGACTCCATGTTCGGGATCA	CACCTGTGAAGGTTTGGCG
ABCG1	CGTGCGCTTTGTGCTGTTT	CCACTGTAGGTACGTGGGGAT
UROS	GCCAAGTCAGTGTATGTGGTT	GCAATCCCTTTGTCCTTGAGC
GAL3ST2	CTCAACATCCTCTACCGCTTCG	GGTGTCGTTGGGCATGACTT
